# Displacement Assay in a Polythiophene Sensor System Based on Supramacromolecuar Disassembly-Caused Emission Quenching

**DOI:** 10.3390/s24134245

**Published:** 2024-06-29

**Authors:** Tsukuru Minamiki, Ryosuke Esaka, Ryoji Kurita

**Affiliations:** 1 Health and Medical Research Institute, National Institute of Advanced Industrial Science and Technology (AIST), 1-1-1 Higashi, Tsukuba 305-8566, Ibaraki, Japanr.kurita@aist.go.jp (R.K.); 2 Precursory Research for Embryonic Science and Technology (PRESTO), Japan Science and Technology Agency (JST), 4-1-8 Honcho, Kawaguchi 332-0012, Saitama, Japan; 3 Faculty of Pure and Applied Sciences, University of Tsukuba, 1-1-1 Tennodai, Tsukuba 305-8573, Ibaraki, Japan

**Keywords:** polythiophene, fluorescent sensor, solid-state emission, displacement assay, biogenic amines, amino acids, aminoglycosides

## Abstract

Exploring new methodologies for simple and on-demand methods of manipulating the emission and sensing ability of fluorescence sensor devices with solid-state emission molecular systems is important for realizing on-site sensing platforms. In this regard, although conjugated polymers (CPs) are some of the best candidates for preparing molecular sensor devices owing to their luminescent and molecular recognition properties, the development of CP-based sensor devices is still in its early stages. In this study, we herein propose a novel strategy for preparing a chemical stimuli-responsive solid-state emission system based on supramacromolecular assembly-induced emission enhancement (SmAIEE). The system was spontaneously developed by mixing only the component polymers (i.e., polythiophene and a transient cross-linking polymer). The proposed strategy can be applied to the facile preparation of molecular sensor devices. The analyte-induced fluorescent response of polythiophene originated from the dynamic displacement of the transient cross-linker in the polythiophene ensemble and the generation of the polythiophene–analyte complex. Our successful demonstration of the spontaneous preparation of the fluorescence sensor system by mixing two component polymers could lead to the development of on-site molecular analyzers including the determination of multiple analytes.

## 1. Introduction

**Figure 1:**
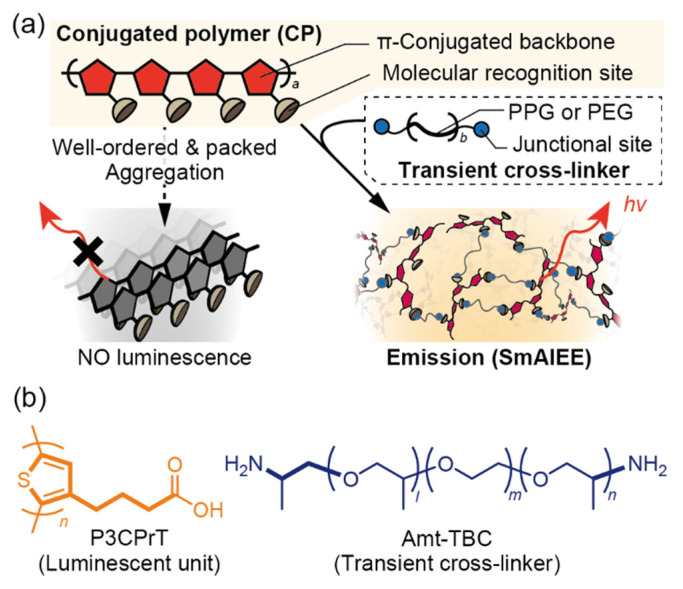
(**a**) Concept of supramacromolecular assembly-induced emission enhancement (SmAIEE) for polythiophene. A solid-state-emission system that could be utilized as a film-type chemical sensor was spontaneously fabricated by complexing a conjugated polymer and a non-conjugated polymer. (**b**) Macromolecular components utilized to prepare a chemical stimuli-responsive solid-state emission system.

Conjugated polymers (CPs) have been extensively researched and developed owing to their interesting features such as ease of processing and unique luminescent properties that reflect their higher-ordered structures [1]. Several CP-based luminescent devices (e.g., light-emitting diodes [2] and light-emitting electrochemical cells [3]) were developed in the early days of organic electronics. Nevertheless, the evolution of these developments remains stagnant despite the widespread use of small molecule-based luminescent devices in the last decade. This problem may be attributed to the strong intra/inter-chain interactions between CP backbones, which intrinsically induce aggregation-induced quenching (Figure 1a) [4]. The molecular insulation technique has been proposed to address this issue of π-conjugated backbones in CPs [5]. However, this approach lacks universality to achieve various applications because it requires complicated molecular designs and synthesis techniques [6]. CP-based sensors have also been prepared to determine several chemical/biological species [7]. Molecular recognition sites wired in series not only enable the amplification of optical responses but also contribute to the identification of complex-structured analytes [8]. Moreover, further development of CP-based sensors is expected for the on-site monitoring of chemical species owing to the excellent processability of polymer materials to obtain solid-state systems. However, although CPs show distinctive molecular recognition behaviors in solvents, few studies have been reported on luminescent sensor devices utilizing CPs because of the aforementioned quenching problems in the solid state [9]. Thus, a simple methodology for obtaining CP films without impairing their optical performance and flexible association ability with heteromolecules should be established to improve the utility of CP materials in sensing device applications.

To this end, we focused on the supramolecular assembly-induced emission enhancement (SAIEE) mechanism [10,11]. The luminescent states in SAIEE systems are spontaneously and dynamically tuned by competing reactions between a small molecule-based fluorophore, a cross-linker, and additives through non-covalent interactions. This strategy was recently utilized to prepare stimuli-responsive systems because their properties can be easily developed and tuned without complicated molecular designs and syntheses [12]. More importantly, SAIEE can help solve the aggregation-induced quenching problem in fluorophores, thereby facilitating the realization of solid-state-emission devices and sensors. However, research on the manipulation of emission states in CPs mediated by the self-assembly of multiple molecules is in its nascent stages. To broaden this concept for the preparation of CP-based luminescent devices and sensors, we herein report a solid-state-emissive sensor based on a supramacromolecular assembly-induced emission enhancement (SmAIEE) system that is achieved by mixing only the component polymers (Figure 1a). In this study, we used a simple acid–base interaction between a well-known CP and a non-CP as the driving force to achieve a CP-based emission system (Figure 1b) [13]. Specifically, we utilized polythiophene with a carboxy side chain (P3CPrT) as the luminescent unit of the system. We also employed an isopropylamine-terminated polypropylene/polyethylene triblock copolymer (Amt-TBC) as the transient cross-linker to control the assembly state of P3CPrT. Isopropylamine at both ends of Amt-TBC acted as a junctional site for each carboxy side chain of the thiophene monomer. Furthermore, these functional groups behaved as molecular recognition sites for analytes [14]. This polythiophene ensemble, prepared simply by mixing and drying, enabled the spontaneous fabrication of a fluorescent film sensor for structurally similar biogenic amines and aminoglycosides.

## 2. Materials and Methods

### 2.1. Preparation of Polythiophene-Ensemble Solutions and Their Films

The polythiophene ensemble was composed of two components: the luminescent unit (P3CPrT) and the transient cross-linker (Amt-TBC) (Figure 1b). The details of the utilized reagents and the device preparation are described in Supplementary Materials.

Initially, the selected macromolecular components (P3CPrT and Amt-TBC) were dissolved in the DMSO solvent (Figure S4a). To dissolve a P3CPrT powder into DMSO, the solution was heated at 95 °C for 30 min and then cooled at room temperature for 1 h. The final concentration of the P3CPrT solution was 0.1 wt%. Amt-TBC was also dissolved in the DMSO solvent at concentrations of 0.5, 1, 3, 5, 7, 10, 13, 16, 20, and 25 wt%. Lastly, the DMSO solutions of P3CPrT and Amt-TBC were mixed at a volume ratio of 1:1 (*v*/*v*). Therefore, the final concentrations of the polymers in the mixed solution were as follows: [P3CPrT] = 0.05 wt%, [Amt-TBC] = 0.25, 0.5, 1.5, 2.5, 3.5, 5, 6.5, 8, 10, and 12.5 wt%. The mixed solution was applied to characterize the optical behaviors of the polythiophene-ensemble system in the bulk phase (DMSO). The coexisting weight ratios of Amt-TBC to P3CPrT in the ensemble system were defined as *X*:1. Next, polythiophene-ensemble films were deposited on glass substrates (microwell or micro-hole slide plates) for optical characterizations and fluorescence titration experiments (Figure S4b). Before the deposition process, the substrate was cleaned with pure water. The polythiophene-ensemble film was deposited by drop-casting from the polythiophene-ensemble solution. Then, the drop-casted solution was gradually dried at 25 °C for 2 h and 35 °C for 1 h and subsequently annealed at 45 °C for 3 h under a vacuum to prepare the film.

### 2.2. Acquisition and Analysis of Fluorescence Properties of Polythiophene-Ensemble Films

Fluorescence spectra (1) and images (2) of polythiophene-ensemble films were individually collected in accordance with the following schemes. Note that the applied excitation wavelengths slightly altered in each scheme due to a difference in the characteristics of the utilized irradiation sources.

(1) For the characterization of the spectrographic properties of the prepared film, the fluorescent spectrum of the polythiophene-ensemble film was measured using an optical fiber spectrometer. An excitation light (*λ*_ex_: 475 nm) was irradiated to the film through the optical fiber from a UV light source coupled with a single-band bandpass filter.
(2) To evaluate the association and dissociation between each polymer and analyte, the fluorescent image of the polythiophene-ensemble film was captured using a cooled CCD camera equipped with a longwave-pass filter (>520 nm) at the front of the camera lens under UV irradiation using an LED lamp (*λ*_ex_: 470 nm). The exposure time for the image acquisition in each experiment was 0.5 s. After the image acquisition, the fluorescence image was processed with Image J/Fiji software (https://downloads.imagej.net/fiji/ (accessed on 5 June 2024)) on a Windows PC. In this study, the pixel intensity converted from the acquired image was utilized as the fluorescence intensity. The converted intensity value was normalized as (*I* − *I*_0_)/*I*_0_. Here, *I* and *I*_0_ denote the fluorescence intensity of the polythiophene-ensemble film upon the addition of components (Amt-TBC, TBC, or analytes) and the intensity in the absence of components, respectively. Therefore, an increase in (*I* − *I*_0_)/*I*_0_ meant that the film exhibited a “turn-on” fluorescence enhancement. In contrast, a decrease in (*I* − *I*_0_)/*I*_0_ denoted that the film behaved in a “turn-off” quenching mode. In addition, *I*_pH_-basic denotes the fluorescence intensity of the film upon the addition of water containing TBABr (100 mM) at pH 11.67.

To compare the changes of the fluorescence intensities in the polythiophene-ensemble film upon the addition of each analyte, the obtained signals were normalized as (*I*_0_ − *I*)/*I*_0_. The coexisting weight ratio of Amt-TBC (X) to P3CPrT in the polythiophene ensemble was 105. The wavelength of excitation and collection was 470 nm and >520 nm, respectively.

## 3. Results

### 3.1. Investigation of SmAIEE in a Polythiophene Ensemble

**Figure 2:**
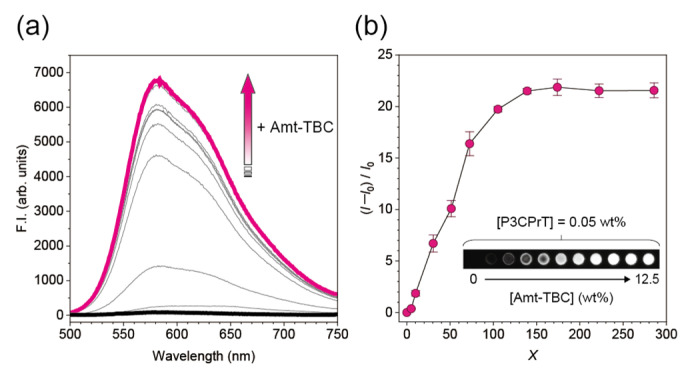
(**a**) Fluorescence spectra of the polythiophene-ensemble film in the absence or presence of Amt-TBC. [P3CPrT] = 0.05 wt% (3.2 × 10^−3^ M/unit), [Amt-TBC] = 0–12.5 wt%. *λ*_ex_: 475 nm. (**b**) Relative change in the fluorescence intensity of the polythiophene-ensemble film upon the addition of Amt-TBC. *λ*_ex_: 470 nm. Inset: Fluorescence image of the polythiophene-ensemble films. The diameter of each film was 1 mm.

Initially, we investigated SmAIEE in a polythiophene ensemble comprising P3CPrT and Amt-TBC. The preparation procedure for the polythiophene ensemble and its film is summarized in the Supplementary Materials. Figure 2 shows the changes in the fluorescence properties of P3CPrT films with increasing weight ratios of Amt-TBC. No emission was obtained from the P3CPrT film without the Amt-TBC additive, whereas the addition of Amt-TBC caused a significant enhancement in the fluorescence intensity and quantum yield of the ensemble films (Figure 2a and Table S3). The fluorescence intensity in the solid phase was also enhanced with increasing levels of Amt-TBC (Figure 2b). Here, the weight ratios of Amt-TBC to P3CPrT in the ensemble were varied as *X*:1. The intensity enhancement plateaued when *X* exceeded 100. At this stage, the molar abundance of the isopropyl amine-terminated group in Amt-TBC was higher than that of the carboxy side chain in P3CPrT. The fluorescence emission of P3CPrT dissolved in dimethyl sulfoxide (DMSO) was also enhanced by the addition of Amt-TBC. This phenomenon was not observed in the control experiment using the same triblock copolymer material without isopropylamine termination (Figure S6). The differences in the fluorescent properties of the ensemble film observed in the presence of diverse electrolytes similarly demonstrated that the selective complexation between the component polymers originated from the acid–base interaction (Figure S8). Moreover, the topography of P3CPrT changed drastically in the absence and presence of Amt-TBC (Figure S9) [15]. These results suggest that the emission enhancement of the polythiophene ensemble was derived from the selective interaction between the functional groups in each polymer, which was assumed to induce the hierarchical organization of P3CPrT and inhibit the aggregation of the conjugated backbone [16]. Therefore, the fluorescence properties of the SmAIEE system may have been influenced by the molecular structure of the functional groups in the component polymers. In fact, the enhancement factor of the fluorescence intensity in polythiophene differed for each alkyl length of the carboxy side chain in polythiophene (Figure S10). These results indicate that various chemical properties of the functional groups in the components can be utilized as parameters for controlling SmAIEE.

### 3.2. Chemical Stimuli Responsiveness in a Polythiophene Ensemble

**Figure 3:**
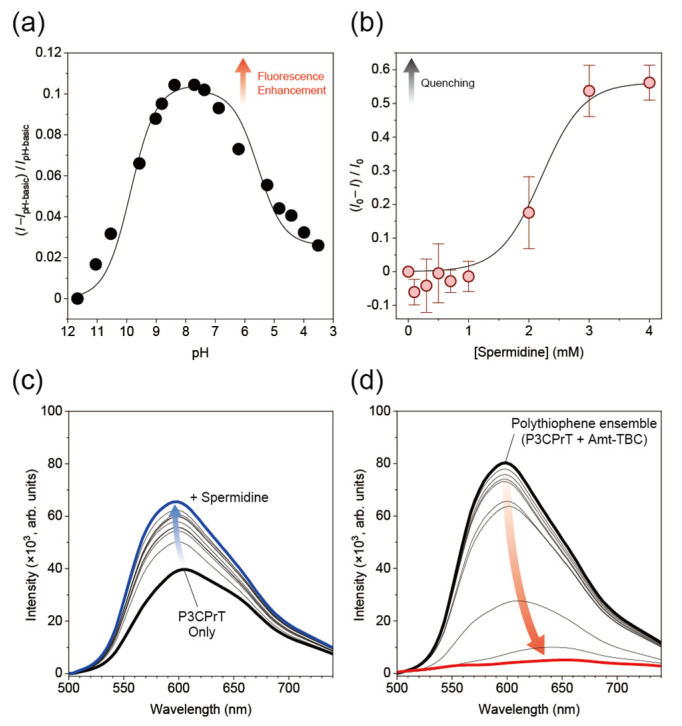
Chemical stimuli responsiveness in the SmAIEE system. (**a**) pH dependency of the fluorescence intensity of the polythiophene-ensemble film with water containing TBABr (100 mM) at 30 °C. The weight ratio of Amt-TBC to P3CPrT (*X*) in the ensemble was 105. (**b**) Changes in the fluorescence intensity of the polythiophene-ensemble film induced by spermidine at various concentrations in HEPES buffer solution (100 mM) with TBABr (100 mM) at pH 7.0 at 30 °C. (**c**,**d**) Fluorescence spectra of P3CPrT (0.05 wt%; 3.2 × 10^−3^ M/unit) in the absence (**c**) or presence (**d**) of Amt-TBC (5 wt%) in DMSO with increasing concentrations of spermidine (0, 1, 3, 5, 7, 10, 30, 50, 70, 100, 300, 500, 700, 1000 μM). *λ*_ex_: 475 nm.

To evaluate the responsiveness of the polythiophene ensemble to chemical stimuli, we inspected the pH dependence of the fluorescence intensity of the SmAIEE system. As mentioned earlier, the SmAIEE system in this study was based on the acid–base interaction between isopropylamine and carboxy groups in each polymer, implying that the protonation/deprotonation of these moieties could affect the formation state of the supramacromolecular assembly (i.e., its fluorescent property). As expected, the fluorescence intensity of the polythiophene-ensemble film depended on the pH of the electrolyte solution (Figure 3a). The pKa values of P3CPrT and Amt-TBC estimated from pH titration were determined to be 5.62 and 9.87, respectively (see Supplementary Materials). These estimated values are comparable with the reported values [17,18]. Moreover, an emission enhancement was observed in the neutral pH range. This finding supports the hypothesis that supramacromolecular assembly formation in this study was mainly induced via hydrogen bonding and/or electrostatic interactions [19,20]. More importantly, the prepared film responded optically to chemical stimulation. Hence, the film could behave as a molecular sensing platform.

The responsiveness of the polythiophene-ensemble film to chemical stimuli prompted us to examine the optical sensing of charged analytes. In particular, the assembled carboxy moieties on the polythiophene wire may have discriminated between the cationic states in the analytes [21,22]. Hence, amine derivatives [23] were employed as model targets to evaluate the molecular recognition ability of the SmAIEE system. The fluorescence intensity of the prepared film gradually decreased with increasing concentrations of spermidine, a typical polyamine (Figure 3b). The sigmoidal curve obtained in the titration experiment suggested that spermidine was recognized through multivalent interactions and/or competitive reactions between each component polymer and the analyte. To investigate the mechanism of the response of the polythiophene ensemble to the analytes, we confirmed the fluorescent property of P3CPrT in the liquid phase upon the addition of spermidine in the presence of Amt-TBC (Figure 3c,d). The fluorescence signal of the polythiophene-ensemble solution decreased with the addition of spermidine. Notably, the presence of Amt-TBC in the P3CPrT solution significantly amplified the fluorescence response of the system to the addition of spermidine. The observed spectral shift in the solution suggested that the fluorescent response of P3CPrT originated from the dynamic displacement of Amt-TBC (i.e., the transient cross-linker) in the polythiophene ensemble and the generation of the P3CPrT–spermidine complex [24]. The DMSO solution of P3CPrT without additives fluoresced weakly (Figure 3c), while the emission of the polythiophene-ensemble solution was almost quenched and slightly red-shifted with the addition of spermidine (Figure 3d). As mentioned previously, the hierarchical arrangement (i.e., the extension of the conjugation length) of the P3CPrT chain was induced by the presence of Amt-TBC. This estimation was supported by the results obtained in this comparative study. In addition, the higher-ordered structure of P3CPrT in the presence of Amt-TBC contributed to the construction of the appropriate inclusion field for polyamine recognition. Overall, fluorescence quenching in the prepared film was triggered by the analyte-induced aggregation of P3CPrT, implying that the origin of the fluorescence response is identical to the displacement mechanism in the liquid phase. Adjustment of the arrangement of the polymer chains appears to be a promising means of manipulating the sensing ability of the system.

### 3.3. Analyte Selectivity in a Polythiophene Ensemble-Based Sensor Film

**Figure 4:**
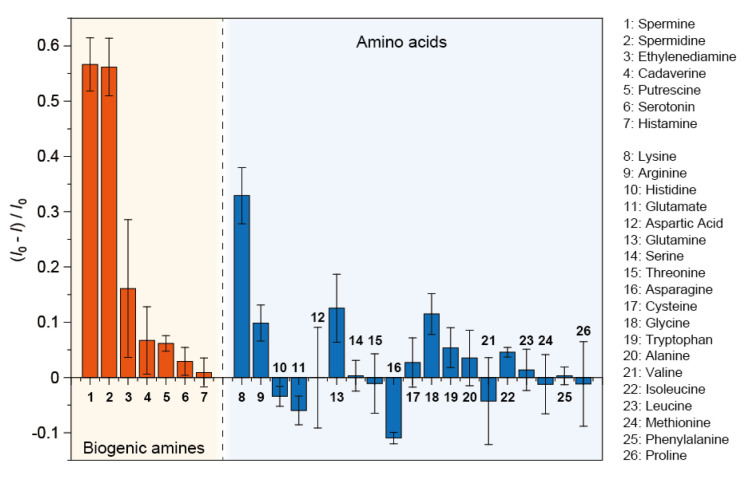
Changes in the fluorescence intensity of the polythiophene-ensemble film upon the addition of biogenic amines or amino acids in HEPES buffer solution (100 mM) with TBABr (100 mM) at pH 7.0 and 30 °C. [Analyte] = 4 mM.

To investigate the selectivity of the prepared film-type sensor, we performed fluorescence titrations for biogenic amines and amino acids (Figure 4). The film responded strongly in the order of the number of cations; the quenching responses to polyamines (spermine and spermidine) were considerably stronger than those to diamines and some amino acids (i.e., lysine). This result suggested that the cation-sensing ability of the carboxy side chain in P3CPrT played a key role in molecular recognition in the prepared system [21,22,25]. By contrast, we observed variable weak responses to the different types of amino acids. This response pattern may be attributed to the presence of Amt-TBC or the electrostatic repulsion between the carboxy moiety of the P3CPrT and amino acids. Furthermore, although the distance between the two NH_2_ groups in lysine and cadaverine was the same, the responses to these analytes were very different. This unique difference may be due to their molecular properties such as mechanical flexibility or hydrophobicity.

**Figure 5:**
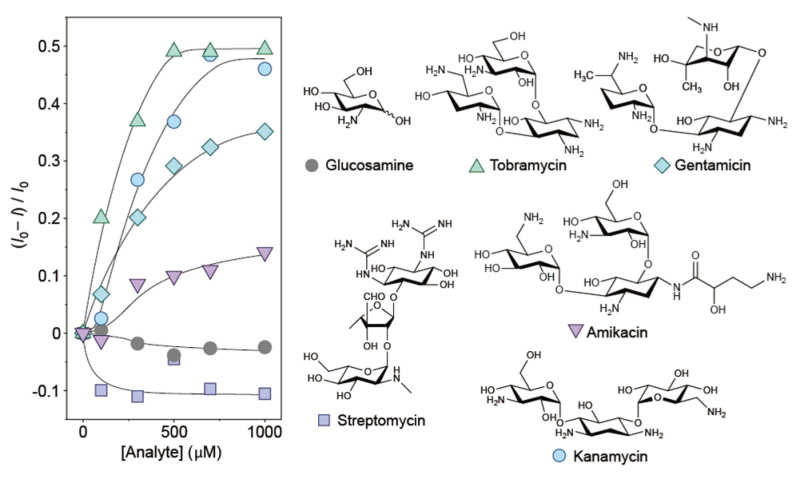
Changes in the fluorescence intensity of the polythiophene-ensemble film with increasing concentrations of glucosamine or aminoglycosides in HEPES buffer solution (100 mM) with TBABr (100 mM) at pH 7.0 and 30 °C.

To further assess the sensing ability of the polythiophene ensemble, we examined the fluorescence detection of aminoglycosides (tobramycin, gentamicin, streptomycin, amikacin, and kanamycin). Aminoglycosides are antibiotics typically used against bacteria [26]. The development of on-site sensing systems for aminoglycoside antibiotics is significant from the viewpoint of the quality management of food products and medicines [27]. Notably, aminoglycosides have sterically complicated structures compared with those of the aforementioned biogenic amines. Hence, these compounds were suitable for investigating the molecular recognition ability of the polythiophene ensemble. Figure 5 summarizes the fluorescence responses of the prepared film to glucosamine and aminoglycosides. Although the prepared film exhibited almost no response to glucosamine, significant responses to aminoglycosides were observed. This difference was attributed to the number of amino groups in the analytes. Furthermore, the origin of the differential responses to aminoglycosides was presumed to be the diversity in the steric conformation and structural isomerism of the amino sites in each compound, indicating that the prepared film was not limited to the discrimination of the chemical properties (e.g., hydrophobicity and electrovalence) of analytes and could also recognize their geometry [28]. The observed association behaviors of P3CPrT to spermidine in DMSO (Figure 3c,d) further suggested that the higher-ordered structure of the CP in the ensemble may have affected the sensing ability of the film toward the structural isomerism of the analytes. The linear range of our system for tobramycin was comparable with or wider than those of the reported sensor devices for this substance (see Supplementary Materials). More importantly, the obtained linear range covered the clinical range of tobramycin. This result indicates that the polythiophene-ensemble system has considerable potential for applications in the on-site determination of aminoglycosides.

### 3.4. Electrolyte Dependency for the Sensing Ability of the Polythiophene Ensemble

**Figure 6:**
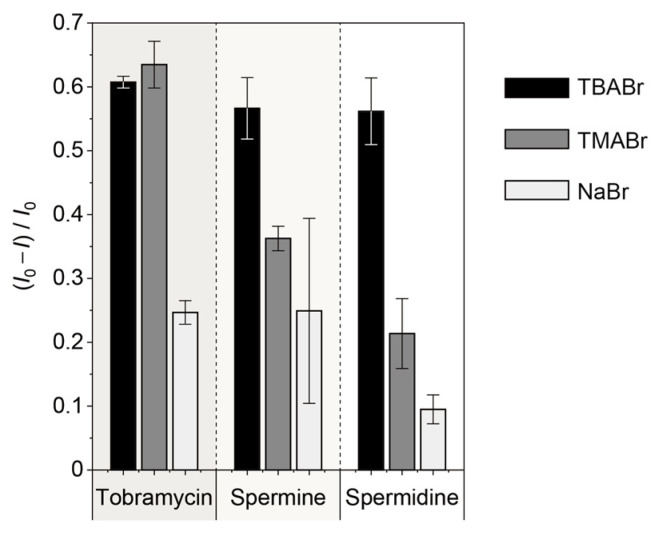
Changes in the fluorescence intensity of the polythiophene-ensemble film as a function of each electrolyte (=TBABr, TMABr, or NaBr; 100 mM). Fluorescent measurements of the film were performed upon the addition of tobramycin, spermine, or spermidine in HEPES buffer solution (100 mM) with electrolyte (100 mM) at pH 7.0 and 30 °C. [Analyte] = 4 mM.

The response of the sensor clearly changed upon altering the electrolytes in the analyte solution (Figure 6). Note that the interferent cations in an analyte solution affect the intermolecular affinity driven by electrostatic interactions. Therefore, the different response behavior of the analyte-induced emission quenching with altering electrolytes might also be attributed to the interaction forces between the component polymers and the analytes as per the Debye shielding effect. In particular, the demonstrated strategy could not only achieve the solid-state emission of CPs but may also have contributed to the acquisition of sensing ability for various guests. In fact, the analyte-induced optical responses were modulated by altering the additives (Figure 6). The obtained cross-reactivity could be tuned by the addition of various interferents. Hence, the simple strategy for the manipulation of cross-reactivity in sensors for various targets could open up an avenue for the development of chemical sensor arrays for high-throughput molecular analyses [29,30].

## 4. Conclusions

In summary, we demonstrated a novel strategy for preparing CP-based solid-state emission systems using only a blend of polymer components. The mixture of P3CPrT and Amt-TBC enabled the spontaneous fabrication of a fluorescent film-type sensor for amine derivatives. The prepared film exhibited distinct fluorescence emissions, and its fluorescence signal was selectively modulated by the addition of analytes. Although we selected P3CPrT and Amt-TBC as representative components to prepare stimuli-responsive fluorescent films in this study, various polymers could be employed depending on the application [31]. Because the proposed displacement assay system possesses high simplicity and universality, we believe that polymer-ensemble systems represent a novel strategy for achieving CP-based luminescent devices and molecular sensing platforms. Further demonstration of these newly proposed polythiophene devices is being conducted in our laboratory.

## Data Availability

Data is contained within the article and Supplementary Materials.

## Supplementary Materials

The following supporting information can be downloaded at https://www.mdpi.com/article/10.3390/s24134245/s1, General information on the experiments, preparation scheme, and optical characterization of the polythiophene ensemble and the optical titrations on the prepared film. References [4,15,17,18,25,32,33,34,35,36,37,38,39] are cited in Supplementary Materials.
