# Supplementary material for: Displacement Assay in a Polythiophene Sensor System Based on Supramacromolecuar Disassembly-Caused Emission Quenching
Source: Sensors (Basel). 2024 Jun 29;24(13):4245. doi: 10.3390/s24134245 (PMC11244502; doi:10.3390/s24134245)
Supplement: Supplementary file 1 [file sensors-24-04245-s001.zip › sensors-3070285-supplementary.pdf]

Supplementary Material

Displacement Assay in a Polythiophene Sensor System Based on Supramacromolecuar Disassembly-Caused Emission Quenching

Tsukuru Minamiki 1,2,*, Ryosuke Esaka 1,3 and Ryoji Kurita 1,3

1 Health and Medical Research Institute, National Institute of Advanced Industrial Science and Technology (AIST), 1-1-1 Higashi, Tsukuba 305-8566, Ibaraki, Japan; erbasso1102@gmail.com (R.E.); r.kurita@aist.go.jp (R.K.)

2 Precursory Research for Embryonic Science and Technology (PRESTO), Japan Science and Technology Agency (JST), 4-1-8 Honcho, Kawaguchi 332-0012, Saitama, Japan

3 Faculty of Pure and Applied Sciences, University of Tsukuba, 1-1-1 Tennodai,

Tsukuba 305-8573, Ibaraki, Japan

Table of Contents

General S3

Preparation of polythiophene-ensemble solutions and their films S10

The effect of the terminal site in TBC on SmAIEE S13

Basic optical properties of polythiophene-ensemble systems S14

FE-SEM observation of polymer films S17

The effect of the side-chain length in P3CAT on SmAIEE S18

The determination of the pK_a value of polythiophene-ensemble film S19

Fluorescence titrations on the polythiophene-ensemble film S21

Comparison of the performance of the fabricated sensor with the reported ones S53

References S54

General

All reagents and solvents (Table S1) employed for this study were used as received. The pure water was served from a Milli-Q water purification system (18 MΩ cm at 25 ºC).

Table S1. Reagents and solvents.

Reagent	Abbreviation	CAS No.	[a] Supplier, [b] product code, and [c] grade
Poly[3-(3-carboxypropyl)thiophene-2,5-diyl] ($M_w \approx$ 15,000–25,000)	P3CPrT	-	[a] Rieke Metals, LLC, [b] 4030, [c] Regioregularity: 84–85%
Poly[3-(4-carboxybutyl)thiophene-2,5-diyl] ($M_w \approx$ 30,000–40,000)	P3CBT	-	[a] Rieke Metals, LLC, [b] 4031
Poly[3-(5-carboxypentyl)thiophene-2,5-diyl] ($M_w \approx$ 55,000–65,000)	P3CPeT	-	[a] Rieke Metals, LLC, [b] 4032
Poly[3-(6-carboxyhexyl)thiophene-2,5-diyl] ($M_w \approx$ 50,000–60,000)	P3CHT	-	[a] Rieke Metals, LLC, [b] 4033
O,*O*'-Bis(2-aminopropyl) polypropylene glycol-*block*-polyethylene glycol-*block*-polypropylene glycol ($M_w \approx$ 2,000)[1]	Amt-TBC	65605-36-9	[a] Huntsman Petrochemical, LLC, [b] Jeffamine® ED-2003
Poly(propylene glycol)-*block*-poly(ethylene glycol)-block-poly(propylene glycol) ($M_w \approx$ 2,000)	TBC	9003-11-6	[a] BASF, [b] Pluronic® 10R5
Cytop (amorphous perfluoropolymer)	-	-	[a] AGC Inc., [b] CTX-809SP2
Heptacosafluorotributylamine	-	311-89-7	[a] AGC Inc., [b] CT-solv.180
Dimethyl sulfoxide	DMSO	67-68-5	[a] FUJIFILM Wako Pure Chemical Corporation, [b] 045-24511, [c] 99.5+%
Sodium hydroxide	-	1310-73-2	[a] FUJIFILM Wako Pure Chemical Corporation, [b] 195-13775, [c] 97.0+%
Hydrochloric acid	-	7647-01-0	[a] FUJIFILM Wako Pure Chemical Corporation, [b] 087-01076, [c] 47.0–49.0%

Hydrobromic acid	HBr	10035-10-6	[a] FUJIFILM Wako Pure Chemical Corporation, [b] 084-01042, [c] 35.0–37.0%
Tetrabutylammonium hydroxide	TBAOH	2052-49-5	[a] Tokyo Chemical Industry Co., Ltd., [b] T1685, [c] 40% in Water
Sodium bromide	NaBr	7647-15-6	[a] FUJIFILM Wako Pure Chemical Corporation, [b] 193-01505, [c] 99.5+%
Tetrabutylammonium bromide	TBABr	1643-19-2	[a] Tokyo Chemical Industry Co., Ltd., [b] T0054, [c] >98.0%
Tetramethylammonium bromide	TMABr	64-20-0	[a] Tokyo Chemical Industry Co., Ltd., [b] T0135, [c] >97.0%
2-[4-(2-Hydroxyethyl)-1-piperazinyl]ethanesulfonic acid	HEPES	7365-45-9	[a] Dojindo Laboratories, [b] GB10, [c] >99.0%
Ethylenediamine dihydrochloride		333-18-6	[a] Aldrich, [b] 195804-100G, [c] 98%
1,4-Diaminobutane dihydrochloride (putrescine)		333-93-7	[a] Tokyo Chemical Industry Co., Ltd., [b] D0081, [c] 98+%
1,5-Diaminopentane dihydrochloride (cadaverine)		1476-39-7	[a] Tokyo Chemical Industry Co., Ltd., [b] D0099, [c] 98+%
Spermidine trihydrochloride		334-50-9	[a] Aldrich, [b] 85578-1G, [c] 99.5+%
Spermine tetrahydrochloride		306-67-2	[a] Sigma, [b] S1141-1G, [c] BioReagent for molecular biology
Histamine dihydrochloride		56-92-8	[a] Tokyo Chemical Industry Co., Ltd., [b] H0146, [c] >98.0%
L-Tryptophan	Trp	73-22-3	[a] Tokyo Chemical Industry Co., Ltd., [b] T0541, [c] >98.5%
5-Hydroxytryptamine hydrochloride (serotonin)		153-98-0	[a] FUJIFILM Wako Pure Chemical Corporation, [b] 321-42341, [c] 97+%
D-(+)-Glucosamine hydrochloride	GluA	66-84-2	[a] Tokyo Chemical Industry Co., Ltd., [b] G0044, [c] >98.0%

Kanamycin monosulfate	KanM	25389-94-0	[a] Tokyo Chemical Industry Co., Ltd., [b] K0047, [c] >94.0%
Streptomycin sulfate	StreM	3810-74-0	[a] Tokyo Chemical Industry Co., Ltd., [b] S0834, [c] >95.0%
Gentamicin sulfate	GenM	1405-41-0	[a] Tokyo Chemical Industry Co., Ltd., [b] G0383
Tobramycin sulfate	TobM	-	[a] Aldrich, [b] T1783-100MG
Amikacin disulfate	AM	39831-55-5	[a] Aldrich, [b] A1774-250MG
L-(+)-Lysine dihydrochloride	Lys	657-26-1	[a] Tokyo Chemical Industry Co., Ltd., [b] L0131, [c] >98.0%
L-(+)-Arginine hydrochloride	Arg	1119-34-2	[a] Tokyo Chemical Industry Co., Ltd., [b] A0528, [c] >98.0%
L-Histidine hydrochloride monohydrate	His	5934-29-2	[a] Tokyo Chemical Industry Co., Ltd., [b] H0150, [c] >98.0%
L-Aspartic acid	Asp	56-84-8	[a] Tokyo Chemical Industry Co., Ltd., [b] A0546, [c] >99.0%
L-Glutamic acid	Glu	56-86-0	[a] Tokyo Chemical Industry Co., Ltd., [b] G0059, [c] >99.0%
L-Serine	Ser	56-45-1	[a] Tokyo Chemical Industry Co., Ltd., [b] S0035, [c] >99.0%
L-(-)-Threonine	Thr	72-19-5	[a] Tokyo Chemical Industry Co., Ltd., [b] T0230, [c] >99.0%
L-Asparagine monohydrate	Asn	5794-13-8	[a] Tokyo Chemical Industry Co., Ltd., [b] A0542, [c] >99.0%
L-Glutamine	Gln	56-85-9	[a] Tokyo Chemical Industry Co., Ltd., [b] G0063, [c] >99.0%
Glycine	Gly	56-40-6	[a] Tokyo Chemical Industry Co., Ltd., [b] G0099, [c] >99.0%

L-Alanine	Ala	56-41-7	[a] Tokyo Chemical Industry Co., Ltd., [b] A0179, [c] >99.0%
L-Valine	Val	72-18-4	[a] Tokyo Chemical Industry Co., Ltd., [b] V0014, [c] >98.0%
L-Isoleucine	Ile	73-32-5	[a] Tokyo Chemical Industry Co., Ltd., [b] I0181, [c] >98.0%
L-Leucine	Leu	61-90-5	[a] Tokyo Chemical Industry Co., Ltd., [b] L0029, [c] >99.0%
L-Methionine	Met	63-68-3	[a] Tokyo Chemical Industry Co., Ltd., [b] M0099, [c] >99.0%
L-Phenylalanine	Phe	63-91-2	[a] Tokyo Chemical Industry Co., Ltd., [b] P0134, [c] >98.0%
L-Cysteine Hydrochloride Monohydrate	Cys	7048-04-6	[a] Tokyo Chemical Industry Co., Ltd., [b] C0517, [c] >99.0%
L-Proline	Pro	147-85-3	[a] Tokyo Chemical Industry Co., Ltd., [b] P0481, [c] >99.0%

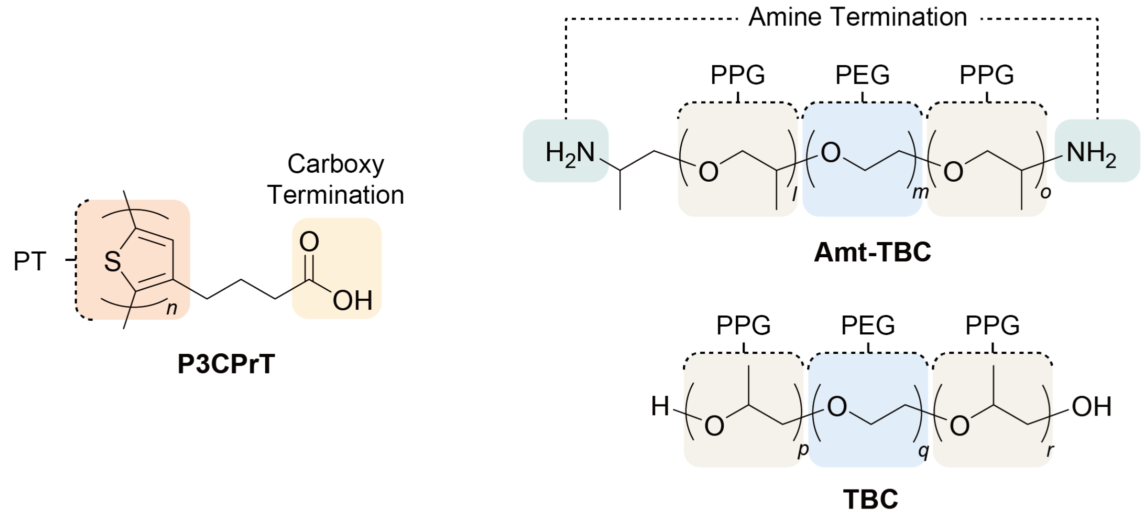

Figure S1 Structures of the utilized polymers for demonstrating the construction of a supramacromolecular assembly in this study.

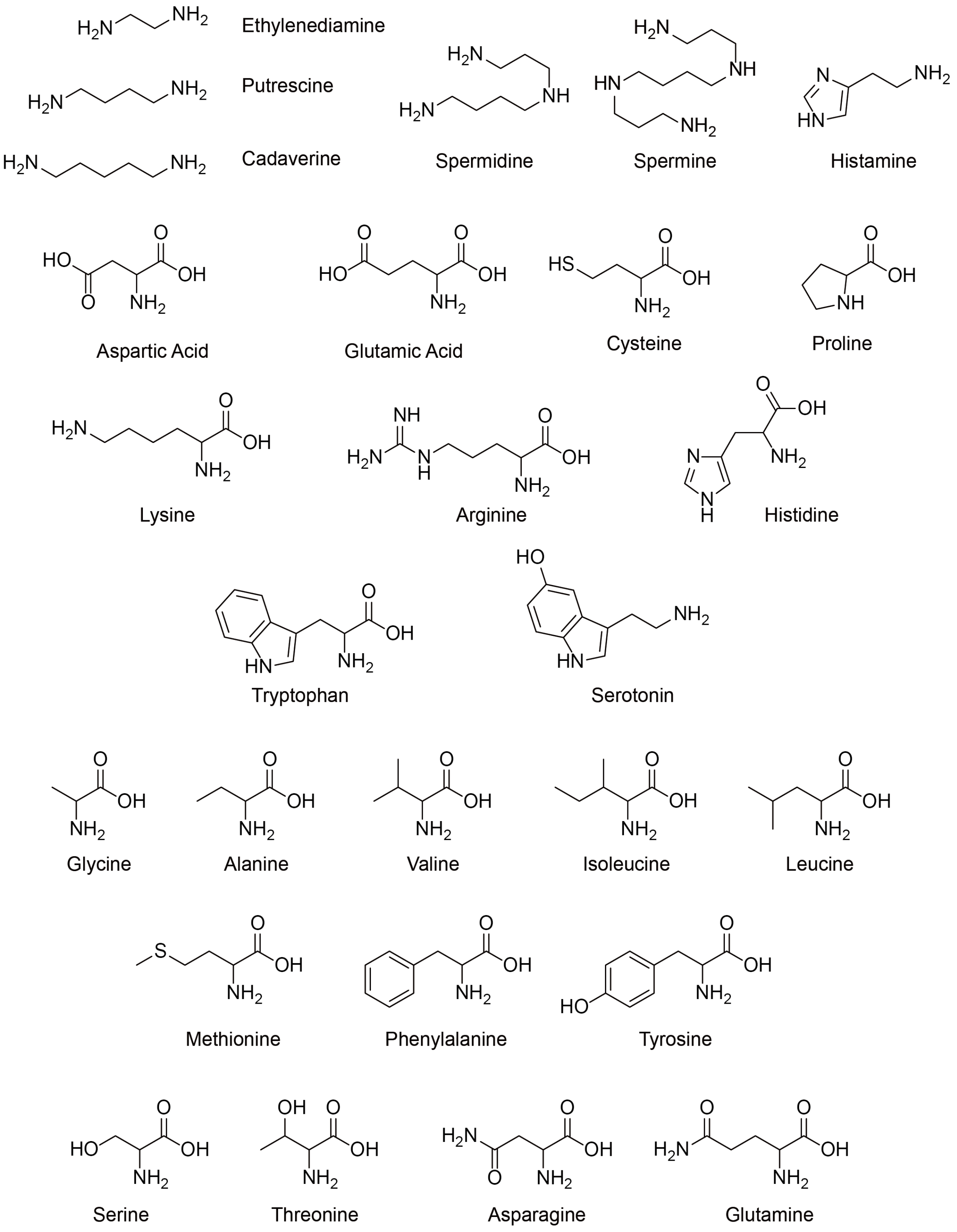

Figure S2 Chemical structures of biogenic amines and amino acids as the analyte species.

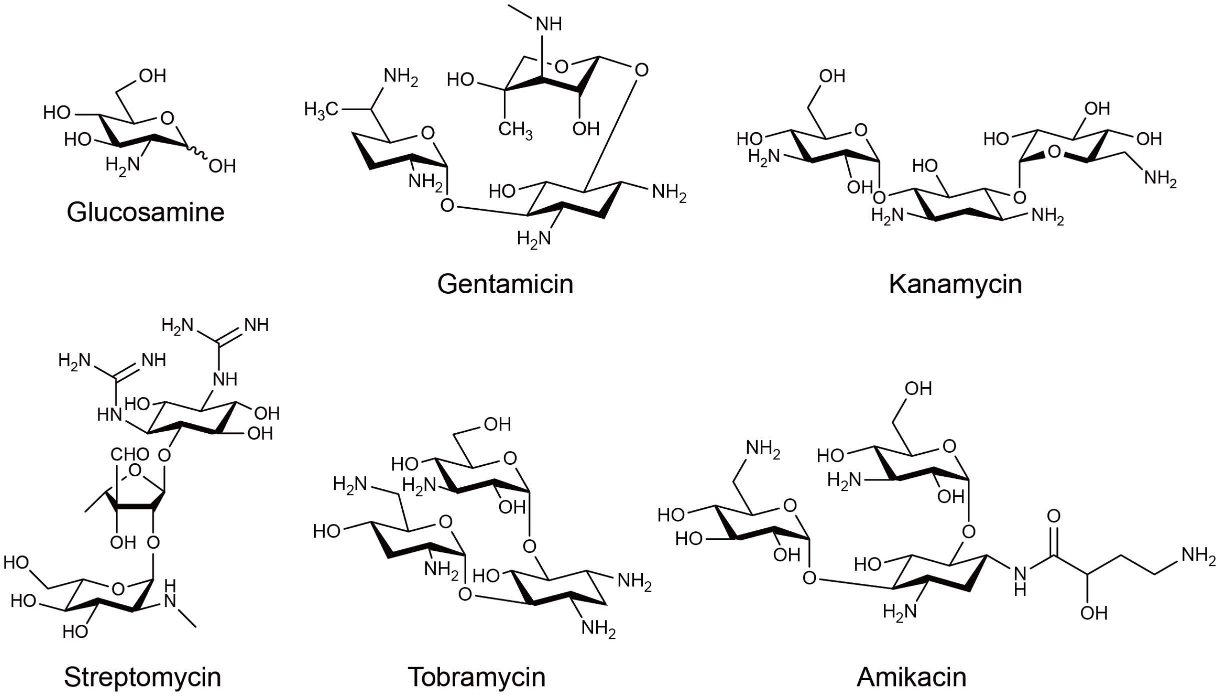

Figure S3 Chemical structures of glucosamine and aminoglycoside antibiotics.

Table S2. Instruments.

Instrument	[a] Brand and [b] type	Condition
Cooling CCD-based image scanner system	[a] AISHIN SEIKI Co. Ltd., [b] LumiVisionPro 400EX	λ_{ex}: 470 nm
Scanning electron microscope (SEM)	[a] Hitachi High-Tech Corp., [b] S-4800II	
UV-visible spectrophotometer	[a] Hitachi Corp., [b] SPM-9700	
Fluorescence spectrophotometer	[a] Thermo Fisher Scientific, Inc., [b] ND-3300 (NanoDrop 3300 Fluorospectrometer)	
Optical fiber spectrometer / Light source	[a] Ocean Optics, Inc., [b] QE65000 / [a] Ocean Optics, Inc., [b] LS-1	λ_{ex}: 475 nm
Microwell slide plate (diameter of each well: 1 mm)	[a] Matsunami Glass Ind., Ltd., [b] S3399E8	
Micro-hole slide plate (diameter of each hole: 4 mm)	[a] Matsunami Glass Ind., Ltd., [b] TF2404	
Hot dry bath equipment	[a] AS ONE Corp., [b] HDB-1N	
Hotplate stirrer equipment	[a] AS ONE Corp., [b] DP-2S	
Vacuum sample drying oven	[a] Ishii Laboratory Works Co., Ltd., [b] HD-15HG	
Vacuum pump	[a] ULVAC KIKO Inc., [b] GCD-051X	
Vortex mixer	[a] AS ONE Corp., [b] MVM-10	
pH meter	[a] HORIBA, Ltd., [b] D-71 LAQUAact Handheld Meter	

Preparation of polythiophene-ensemble solutions and their films

Drastic evaporation of the polythiophene-ensemble solution might induct the heterogeneous crystallization of polythiophene because of a distinct difference in the solubility of each polymer to the polar solvent (i.e., DMSO).[2] Based on this concern, the drop-casted solution was gradually dried at 25°C for 2 h, 35°C for 1 h, and then subsequently annealed at 45°C for 3 h under vacuum for preparing the film. As expected, the crystallization of P3CPrT was partially progressed after the drastic evaporation treatment for the casted solution (45°C for 7 h, *in vacuo*) (Figure S5(a)(b)). The result showed that the drastic evaporation caused the depression and dispersion of fluorescent intensities (Figure S5(c)). This might be derived from aggregation-caused quenching of the polythiophene main-chain by the preferential and hierarchical crystallization of P3CPrT.[3,4] In fact, the obtained fluorescence intensities and their coefficients of variation (CV) were improved with alteration in the drying process (average of the fluorescence intensity: 12.4k → 35.5k, CV: 14.2% → 3.1%). Judging from these results, we employed the gradual drying process as the preparation scheme for fluorescence films with high uniformity and reproducibility.

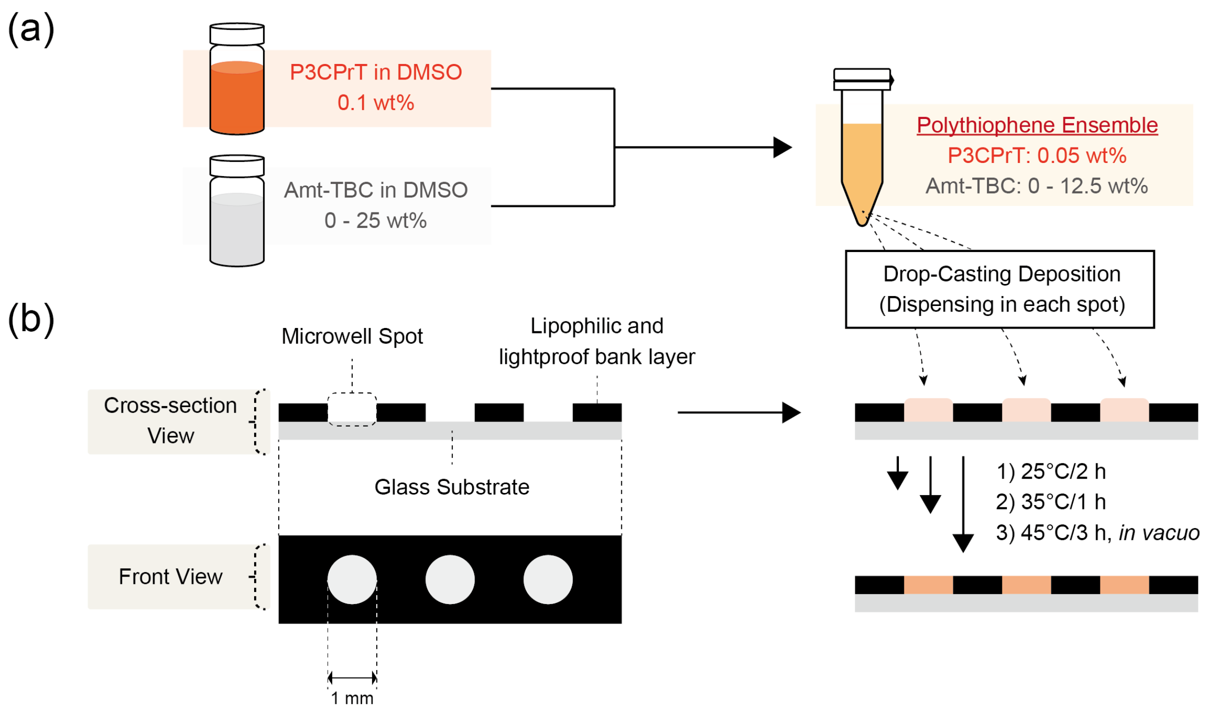

Figure S4 Schematic illustration of the preparation of polythiophene-ensemble solution (a) and film (b).

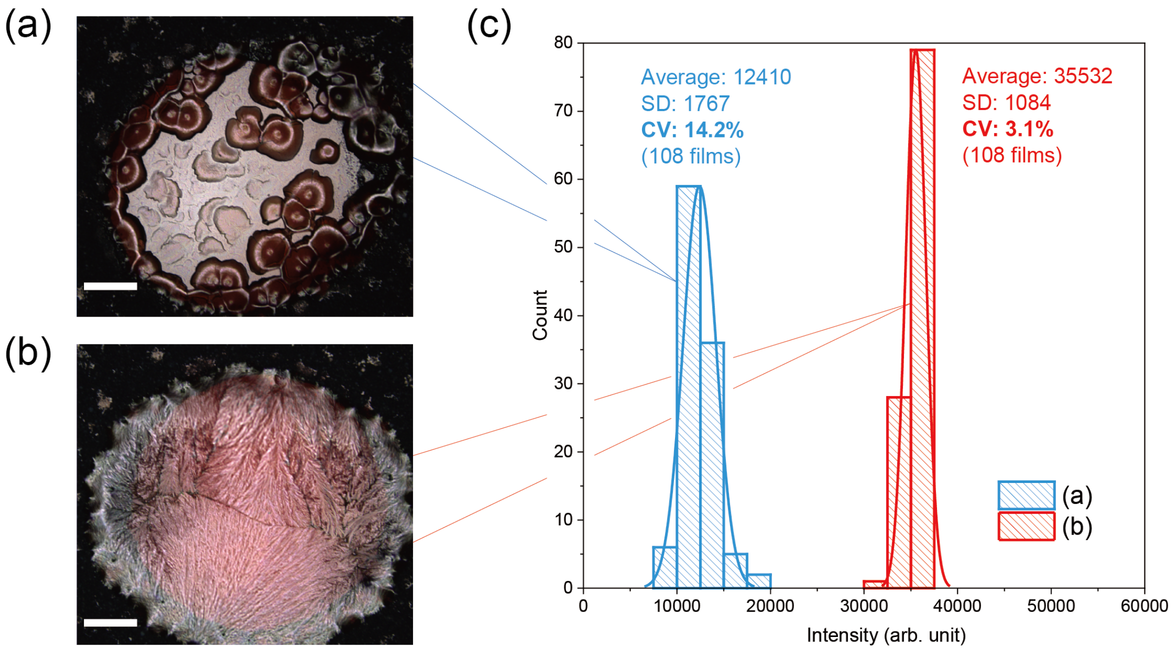

Figure S5 (a,b) Microscopic images of the polythiophene-ensemble film formed by drastic evaporation (45°C for 7 h, *in vacuo*) (a) or gradual drying scheme (cf. Fig. S4) (b). The scale bars indicate 200 μm. (c) Distributions of fluorescent intensities in the polythiophene-ensemble film formed by drastic evaporation (a; blue bars and curve) or gradual drying scheme (b; red bars and curve). The values were measured from 108 films in each condition (exposure time for the image acquisition: 1.0 sec).

The effect of the terminal site in TBC on SmAIEE

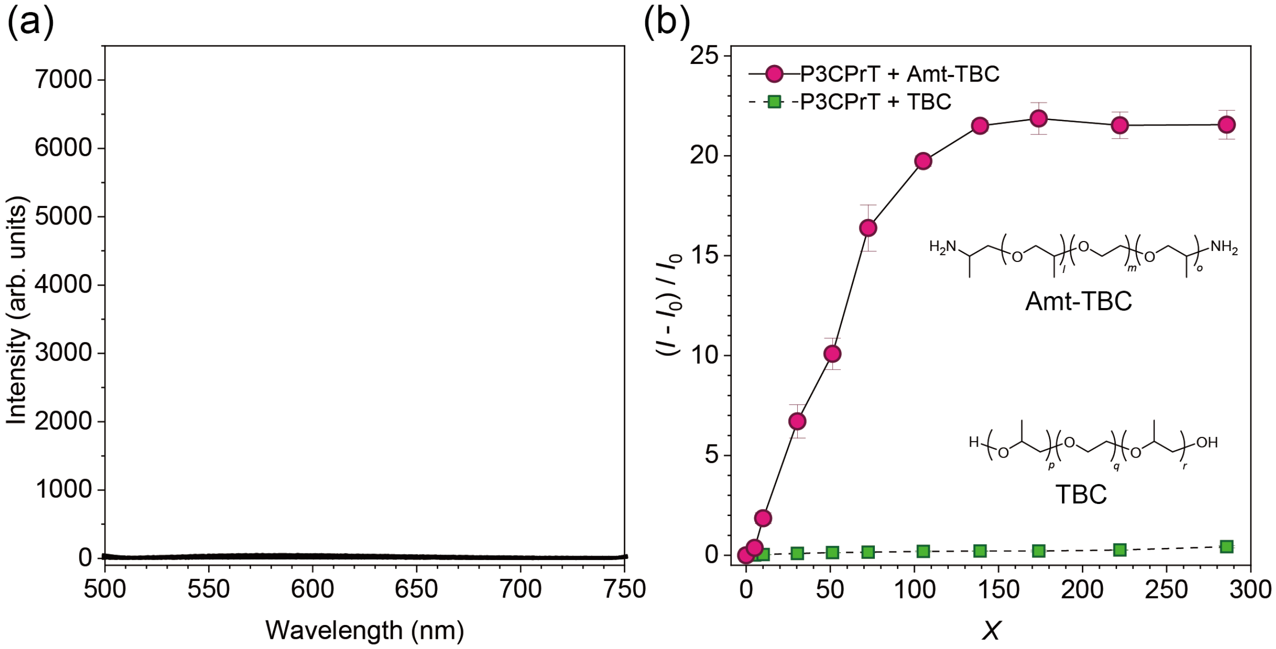

Figure S6 (a) Fluorescence spectra of the P3CPrT film in the absence or the presence of TBC. [P3CPrT] = 0.05 wt% (3.2×10^{-3} M/unit), [TBC] = 0–12.5 wt%. λ_{ex}: 475 nm. (b) The relative change in the fluorescence intensity of the P3CPrT film in the absence or the presence of Amt-TBC (pink circles) or TBC (green squares). [P3CPrT] = 0.05 wt%.

Basic optical properties of polythiophene-ensemble systems

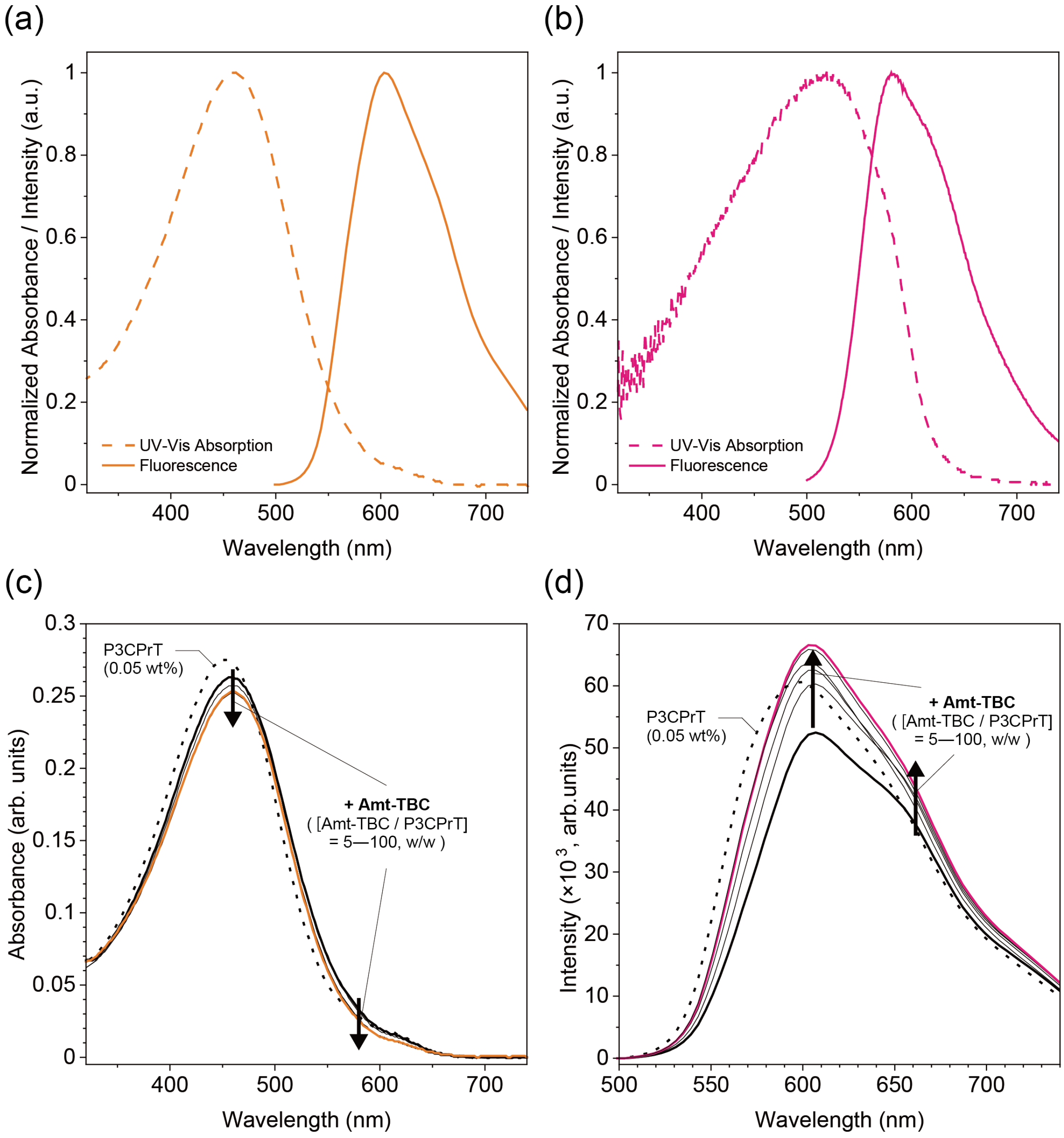

Figure S7 (a) UV-vis absorption (dotted line) and fluorescence (solid line) spectra of the polythiophene ensemble in DMSO. [P3CPrT] = 0.05 wt% (3.2×10^{-3} M/unit), [Amt-TBC] = 5 wt%. (b) UV-vis absorption (dotted line) and fluorescence (solid line) spectra of the polythiophene-ensemble film. [P3CPrT] = 0.05 wt% (3.2×10^{-3} M/unit), [Amt-TBC] = 5 wt%. (c) UV-vis absorption spectra of DMSO solutions containing P3CPrT in the absence (dotted line) or presence (solid line) of Amt-TBC. (d) Fluorescence spectra of DMSO solutions containing P3CPrT in the absence (dotted line) or the presence (solid line) of Amt-TBC.

Table S3. The absolute photoluminescence quantum yields (φ_{PL}) of polythiophene-ensemble systems in the liquid phase ($\varphi_{PL\text{-}L}$) or solid-state ($\varphi_{PL\text{-}S}$).

P3CPrT [wt%]	Amt-TBC [wt%]	X [a]	$\varphi_{PL\text{-}L}$ [%][b,c]	$\varphi_{PL\text{-}S}$ [%][b,d]
0.05	0 [e]	0	9.0	ND [f]
	0.5	10	11.8	0.80
	2.5	51	12.1	2.1
	5.0	105	13.6	2.8

[a] X denotes the function of the coexisting ratio of Amt-TBC to P3CPrT in the ensemble (cf. page S10). [b] All φ_{PL} was measured in an integrating sphere at room temperature. λ_{ex} = 463 nm. [c] The solution samples were prepared in DMSO. [d] The sample films were deposited on a glass substrate. [e] The measured samples consist of P3CPrT only. [f] ND means not determined because a sufficient fluorescence signal was not obtained.

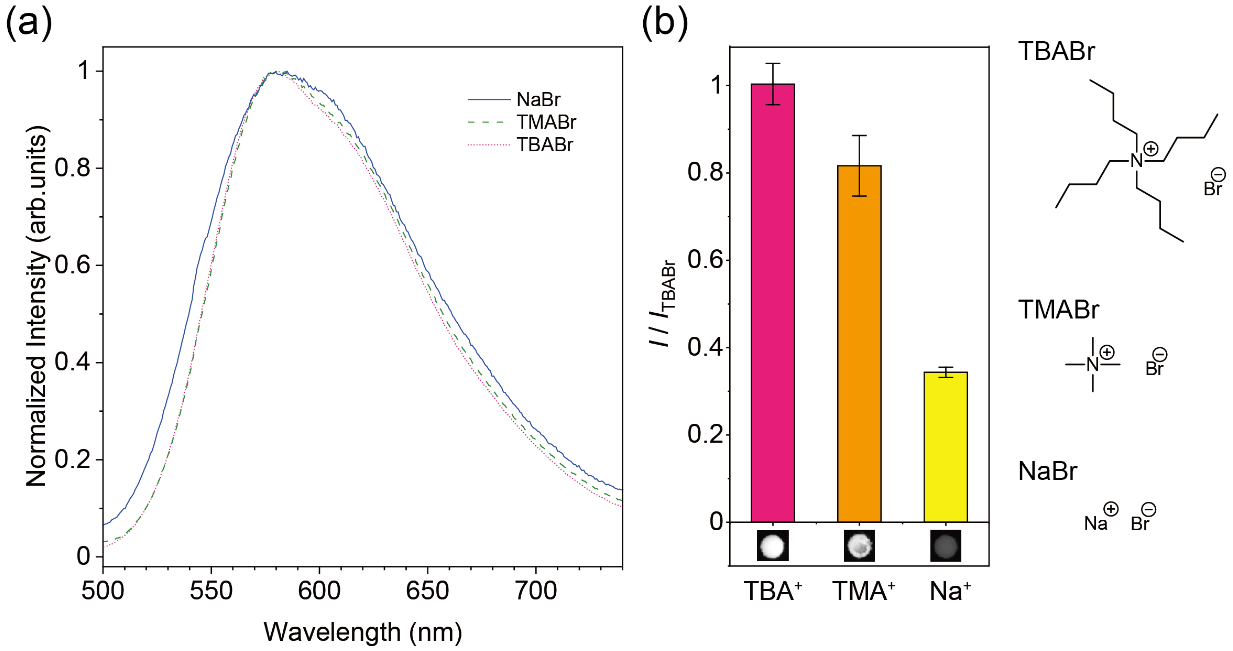

Figure S8 (a) Fluorescence spectra of the polythiophene-ensemble film upon the addition of a HEPES buffer solution (100 mM) with each electrolyte (=TBABr, TMABr, or NaBr; 100 mM) at pH 7.0 at 30°C. [P3CPrT] = 0.05 wt% (3.2×10^{-3} M/unit), [Amt-TBC] = 5 wt%. Although all the measurements exhibited similar energy bands around 580 nm, the spectra were slightly broadened with changing the electrolyte additive from TBA^+ to Na^+. Notably, the shoulder peaks around 540 nm and 610 nm were clearly observed in the spectrum of the NaBr-added film. This suggests that the ordered assembly of the main chain in P3CPrT might be partially occurred by the electrostatic shielding effect with the electrolyte additive.[5,6] (b) Changes in the fluorescence intensity of the polythiophene-ensemble film upon the addition of a HEPES buffer solution (100 mM) with each electrolyte (=TBABr, TMABr, NaBr; 100 mM) at pH 7.0 at 30°C. The intensities of the films (I) are normalized as the function of obtained intensity from the TBABr-added film (I_{TBABr}). The insets show the fluorescent images of the polythiophene-ensemble films (exposure time for the image acquisition: 0.5 sec). The diameters of each film were 1 mm.

FE-SEM observation of polymer films

For the prevention of electrification on the samples, the polymer films were deposited onto a conductive silicon substrate. The film surfaces were shielded by coating the platinum thin-film before the measurement.

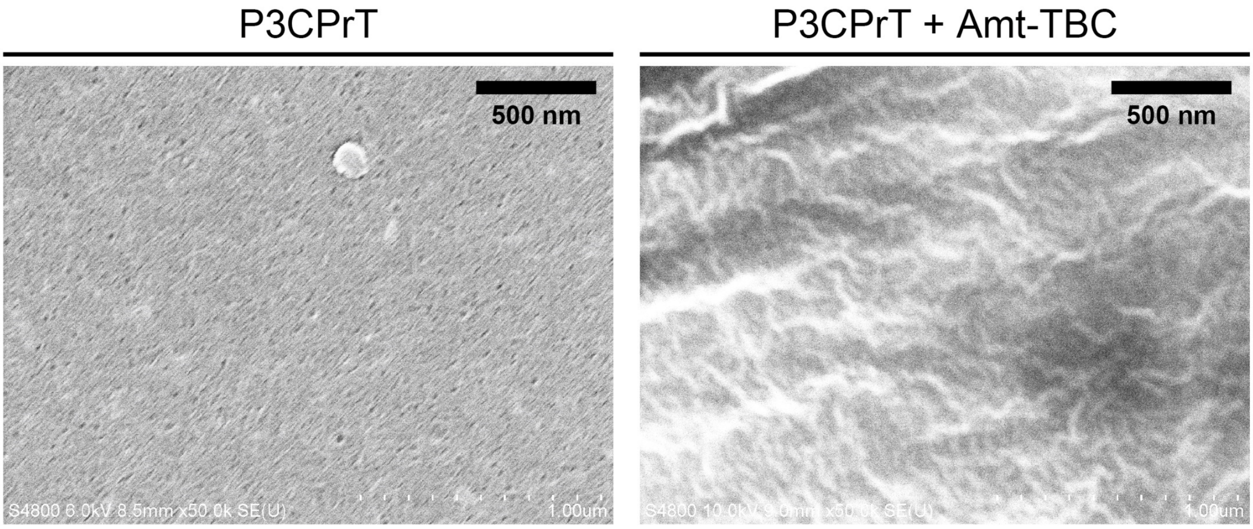

Figure S9 SEM micrographs of P3CPrT films in the absence (left) and the presence (right) of Amt-TBC. [P3CPrT] = 0.05 wt%, [Amt-TBC] = 5 wt%. The coexisting weight ratio of Amt-TBC to P3CPrT (*X*) in the ensemble was 105.

The effect of the side-chain length in P3CAT on SmAIEE

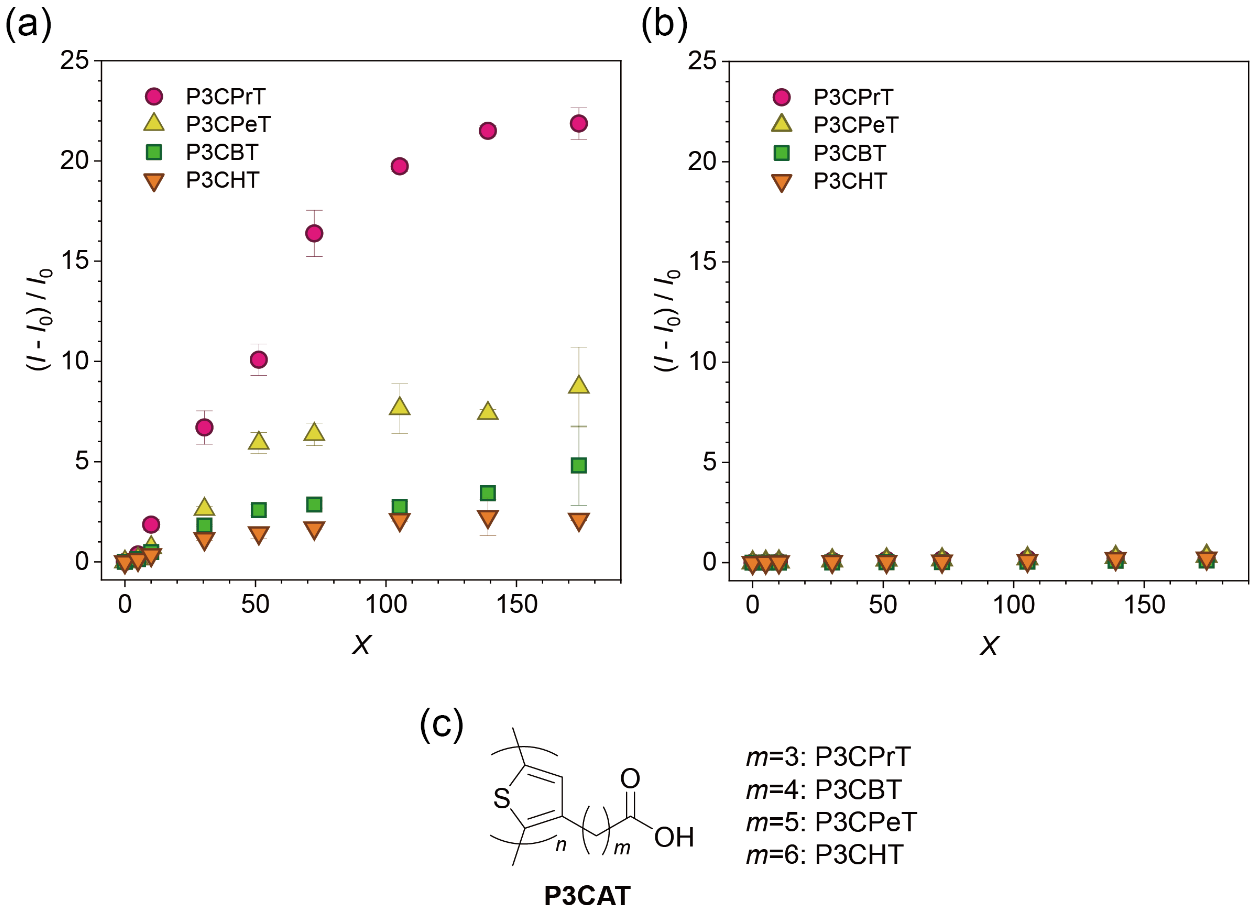

Figure S10 (a) The relative change in the fluorescence intensity of the poly[(3-carboxyalkyl)thiophene] (P3CAT) films in the absence or the presence of Amt-TBC. [P3CAT] = 0.05 wt%, [Amt-TBC] = 5 wt%. (b) The relative change in the fluorescence intensity of the P3CAT films in the absence or the presence of TBC. [P3CAT] = 0.05 wt%, [TBC] = 5 wt%. (c) Chemical structure of the P3CATs series with the different alkyl-length of side-chain.

The determination of the pK_a value of polythiophene-ensemble film

pH-Dependent fluorescent intensities of the polythiophene-ensemble film were obtained. Before dropping water containing TBABr (100 mM) to each film, the pH condition of the solution was controlled by using HBr or TBAOH solution, and then the pH value was measured by a pH meter. The pK_a value could be calculated from the data (Fig. 3a) using the following equation[7]:

$$I = \frac{(I_i + I_{max} K_a [\mathrm{H}^+])}{(1 + K_a [\mathrm{H}^+])}$$

when I is the fluorescent intensity for a particular concentraton of proton; I_i is initial intensity of the titration experiment; I_{max} is the maximum intensity in the titration experiment; K_a is the acid dissociation constant; $[H^+]$ is the concentration of proton. The pK_a profile can be analyzed using nonlinear curve fitting based on the Levenberg-Marquardt algorithm in the OriginPro software on a Windows PC.[8] As a result, two inflection points were obtained in the pH titration curve for the polythiophene-ensemble film (Fig. 3a), meaning that the obtained result reflects the protonation and deprotonation behaviours of the termination structures in P3CPrT (=the carboxylated side-chain) and Amt-TBC (=the aminopropyl end-group). The estimated pK_a values of P3CPrT and Amt-TBC in the solid-state were 5.62 and 9.87, respectively (Table S3). The correlation coefficient (R^2) in the pH titration curve was > 0.94 (Fig. 3a).

Table S4. The pK_a values of reference compounds and the utilized macromolecular components.

Compound	pK_a	Reference
Poly(3-thiophene acetic acid)	4 – 6	*Macromolecules*, 1999, **32**, 3964.[9]
P3CPeT	4.2	*Chem. Commun.*, 2018, **54**, 6907.[10]
Isopropyl amine	10.63	*J. Am. Chem. Soc.*, 1957, **79**, 5441.[10]
Bis(3-aminopropyl) terminated polyethylene glycol (Jeffamine®)	9.72	*Chem. Eur. J.*, 2007, **13**, 9663.[11]
P3CPrT	5.62	Fig. 3a (This study)
Amt-TBC	9.87	Fig. 3a (This study)

Fluorescence titrations on the polythiophene-ensemble film

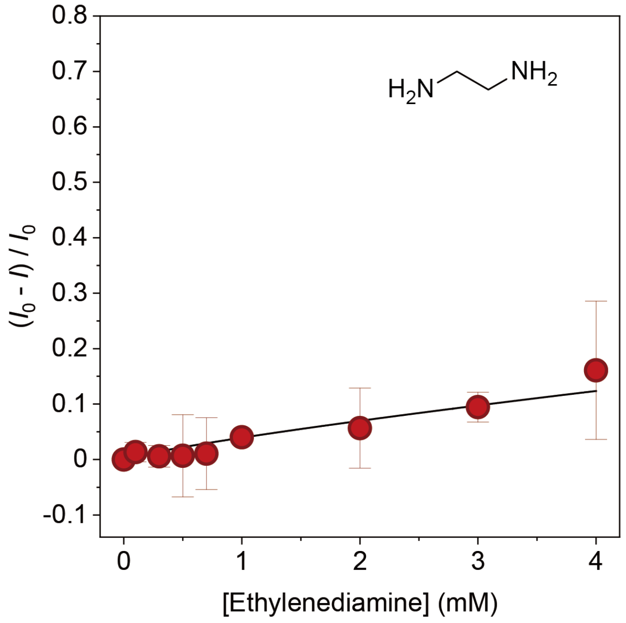

Figure S11 Changes in fluorescence intensity for the polythiophene-ensemble film by ethylenediamine at various concentrations in a HEPES buffer solution (100 mM) with TBABr (100 mM) at pH 7.0 at 30°C.

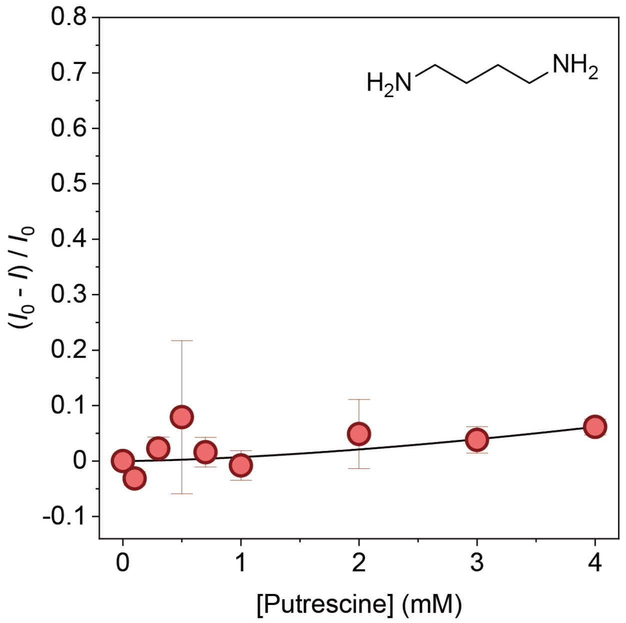

Figure S12. Changes in fluorescence intensity for the polythiophene-ensemble film by putrescine at various concentrations in a HEPES buffer solution (100 mM) with TBABr (100 mM) at pH 7.0 at 30°C.

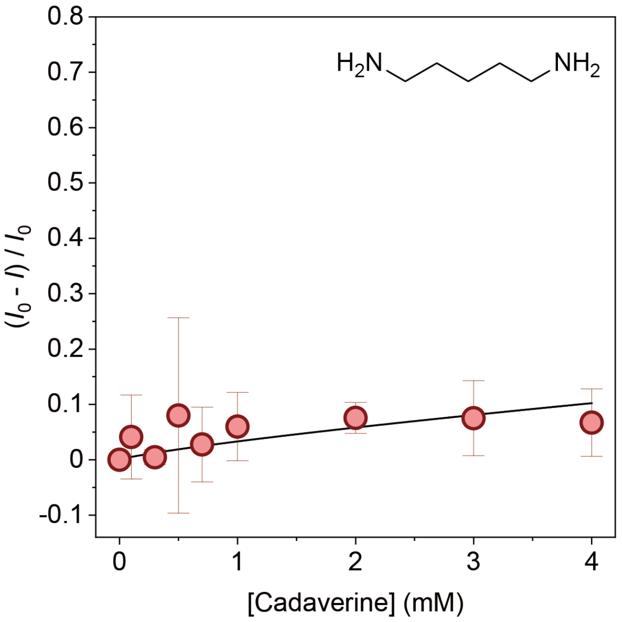

Figure S13 Changes in fluorescence intensity for the polythiophene-ensemble film by cadaverine at various concentrations in a HEPES buffer solution (100 mM) with TBABr (100 mM) at pH 7.0 at 30°C.

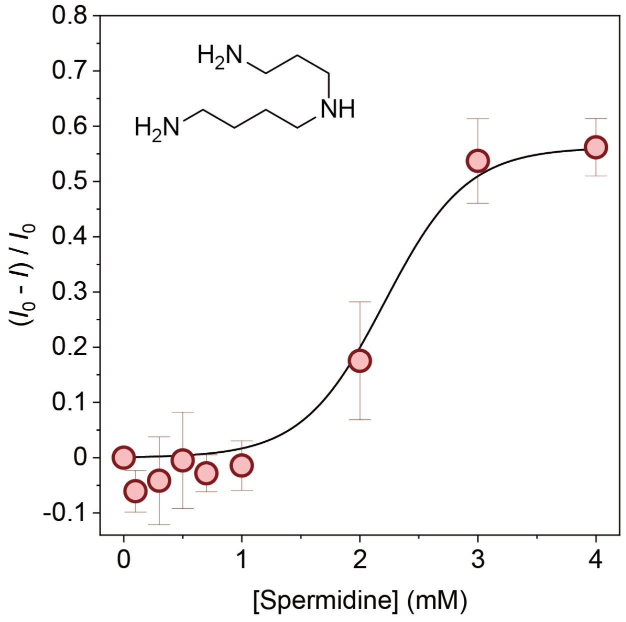

Figure S14 Changes in fluorescence intensity for the polythiophene-ensemble film by spermidine at various concentrations in a HEPES buffer solution (100 mM) with TBABr (100 mM) at pH 7.0 at 30°C.

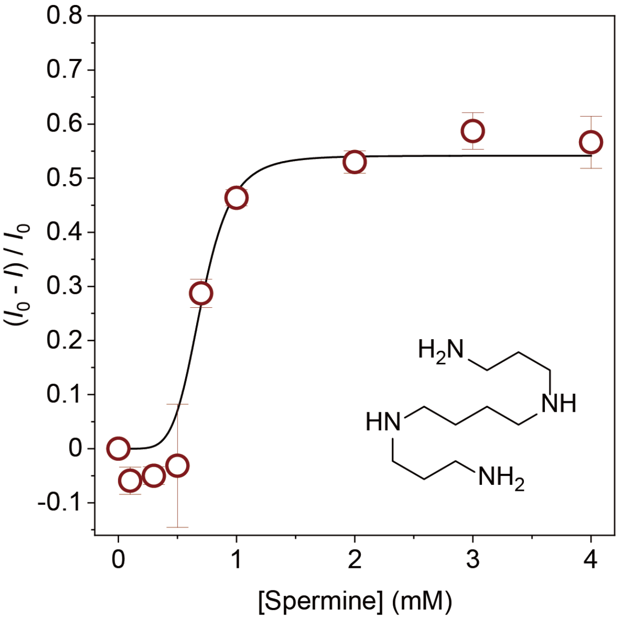

Figure S15 Changes in fluorescence intensity for the polythiophene-ensemble film by spermine at various concentrations in a HEPES buffer solution (100 mM) with TBABr (100 mM) at pH 7.0 at 30°C.

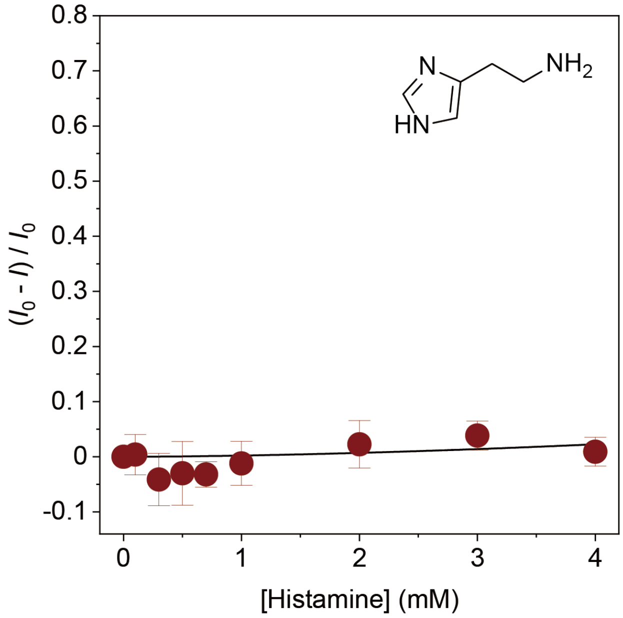

Figure S16 Changes in fluorescence intensity for the polythiophene-ensemble film by histamine at various concentrations in a HEPES buffer solution (100 mM) with TBABr (100 mM) at pH 7.0 at 30°C.

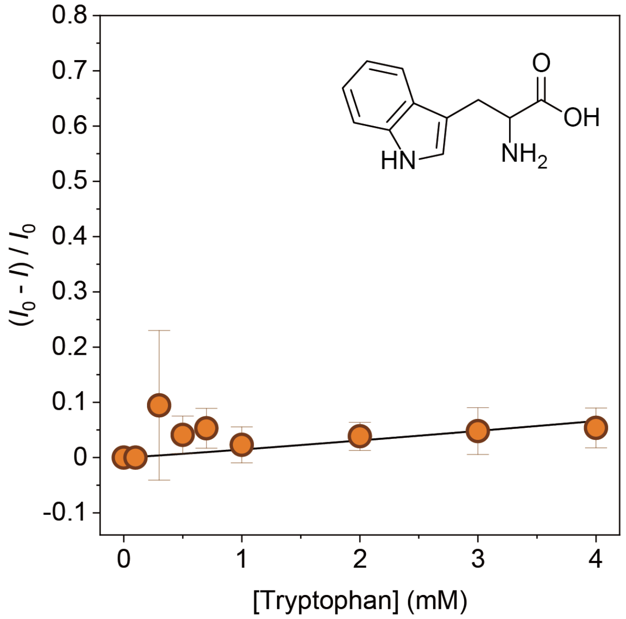

Figure S17 Changes in fluorescence intensity for the polythiophene-ensemble film by tryptophan at various concentrations in a HEPES buffer solution (100 mM) with TBABr (100 mM) at pH 7.0 at 30°C.

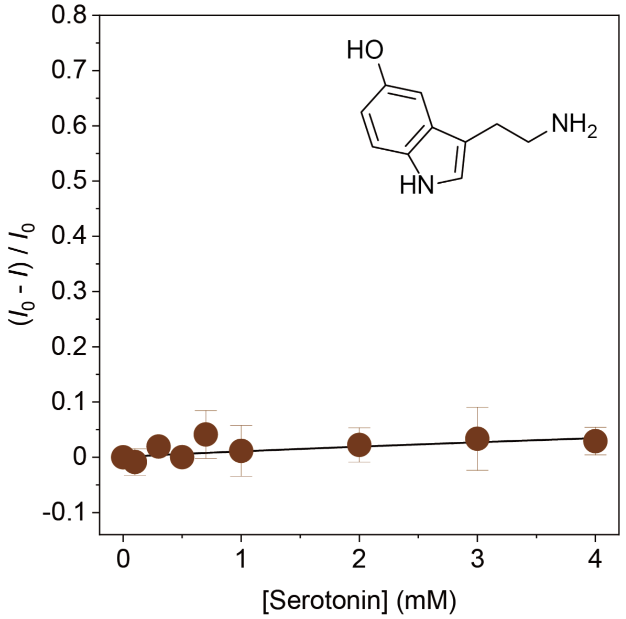

Figure S18 Changes in fluorescence intensity for the polythiophene-ensemble film by serotonin at various concentrations in a HEPES buffer solution (100 mM) with TBABr (100 mM) at pH 7.0 at 30°C.

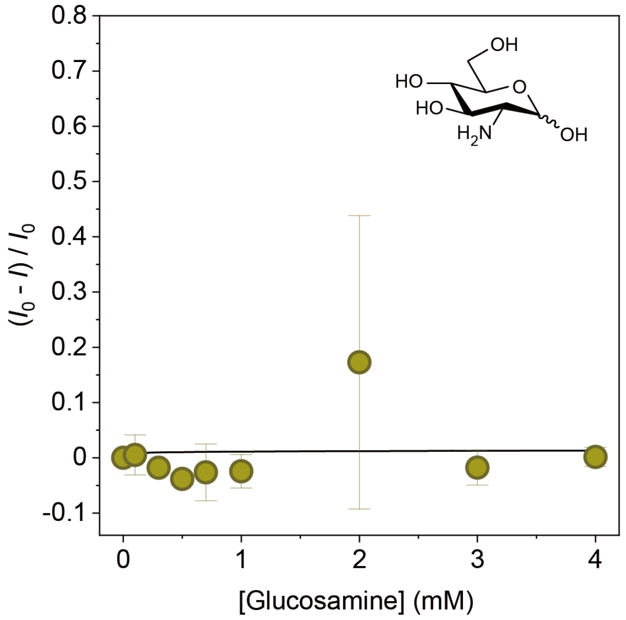

Figure S19 Changes in fluorescence intensity for the polythiophene-ensemble film by glucosamine at various concentrations in a HEPES buffer solution (100 mM) with TBABr (100 mM) at pH 7.0 at 30°C.

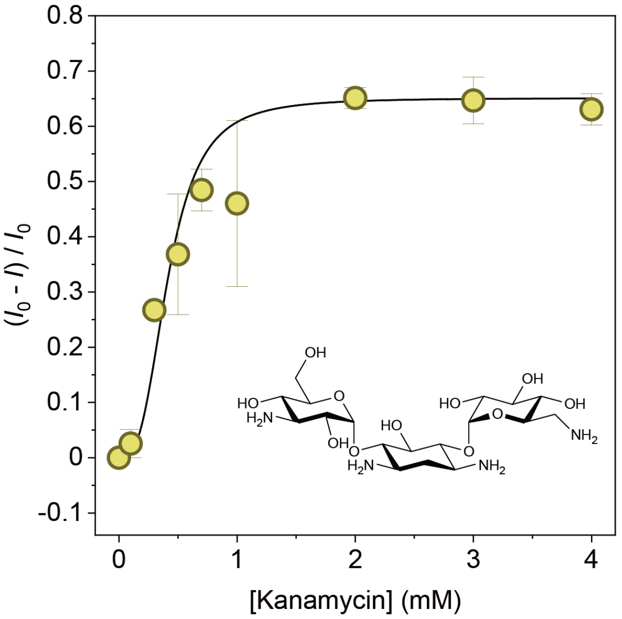

Figure S20 Changes in fluorescence intensity for the polythiophene-ensemble film by kanamycin at various concentrations in a HEPES buffer solution (100 mM) with TBABr (100 mM) at pH 7.0 at 30°C.

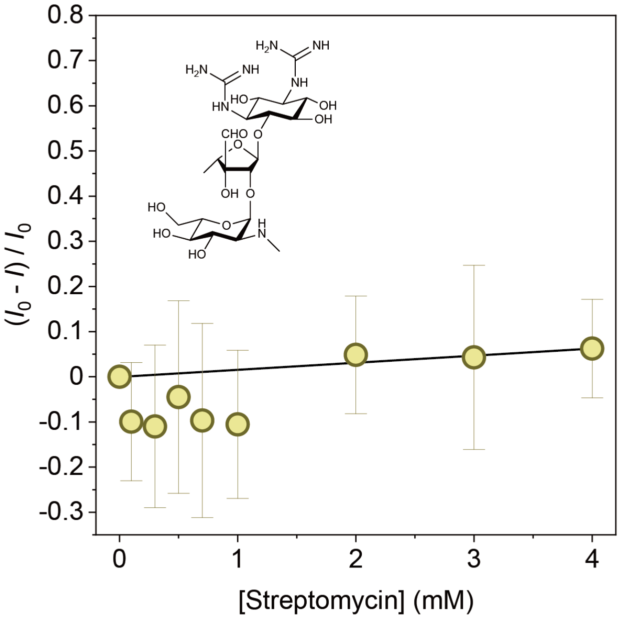

Figure S21 Changes in fluorescence intensity for the polythiophene-ensemble film by streptomycin at various concentrations in a HEPES buffer solution (100 mM) with TBABr (100 mM) at pH 7.0 at 30°C.

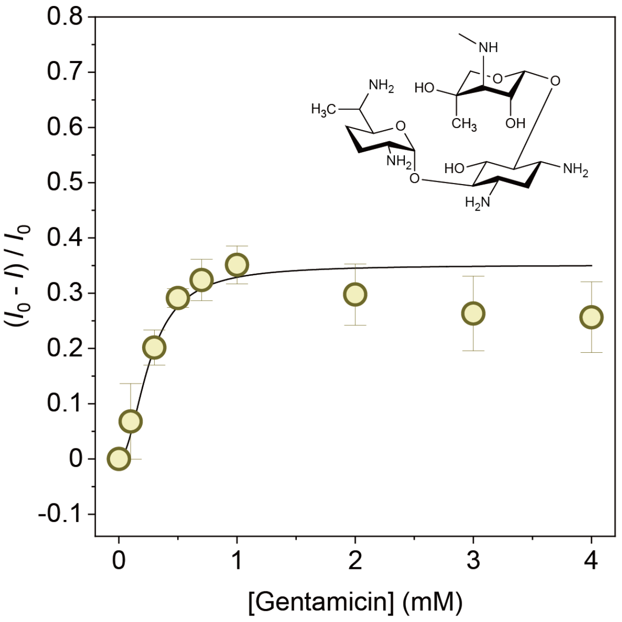

Figure S22 Changes in fluorescence intensity for the polythiophene-ensemble film by gentamicin at various concentrations in a HEPES buffer solution (100 mM) with TBABr (100 mM) at pH 7.0 at 30°C.

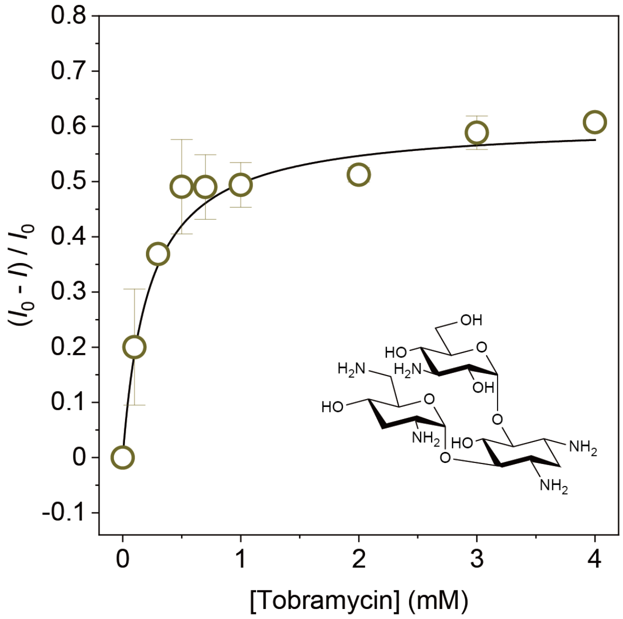

Figure S23 Changes in fluorescence intensity for the polythiophene-ensemble film by tobramycin at various concentrations in a HEPES buffer solution (100 mM) with TBABr (100 mM) at pH 7.0 at 30°C.

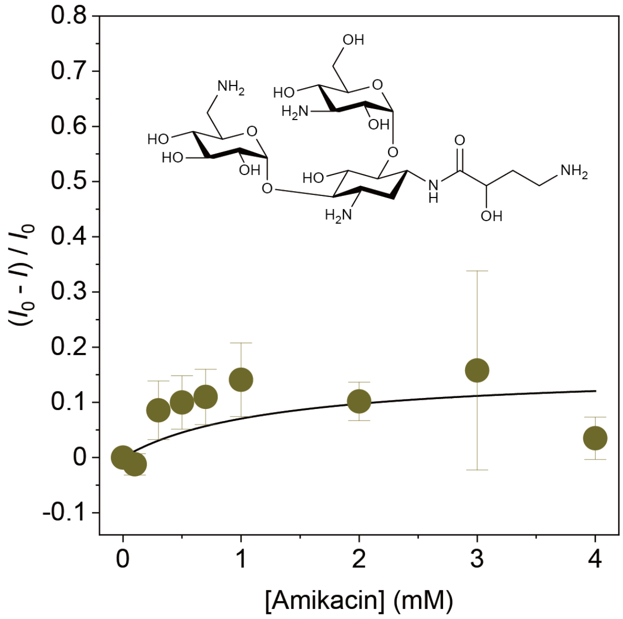

Figure S24 Changes in fluorescence intensity for the polythiophene-ensemble film by amikacin at various concentrations in a HEPES buffer solution (100 mM) with TBABr (100 mM) at pH 7.0 at 30°C.

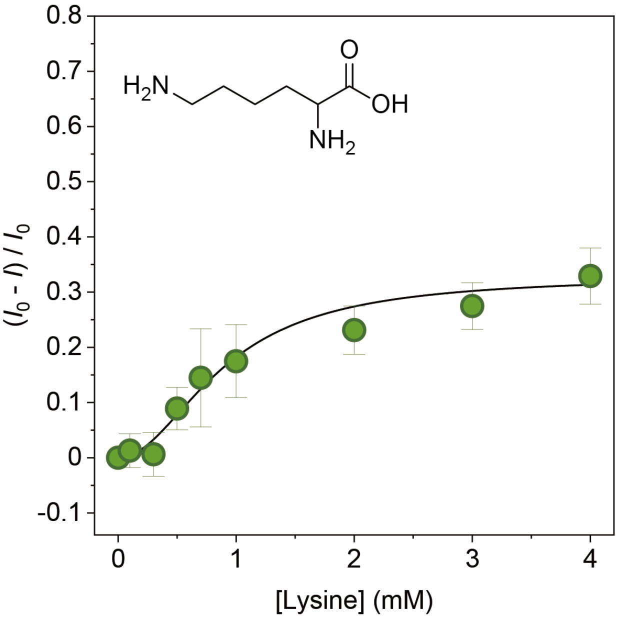

Figure S25 Changes in fluorescence intensity for the polythiophene-ensemble film by lysine at various concentrations in a HEPES buffer solution (100 mM) with TBABr (100 mM) at pH 7.0 at 30°C.

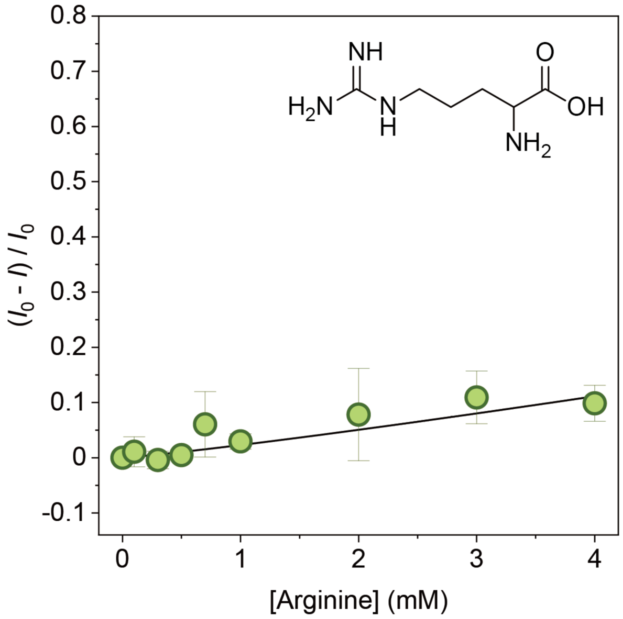

Figure S26 Changes in fluorescence intensity for the polythiophene-ensemble film by arginine at various concentrations in a HEPES buffer solution (100 mM) with TBABr (100 mM) at pH 7.0 at 30°C.

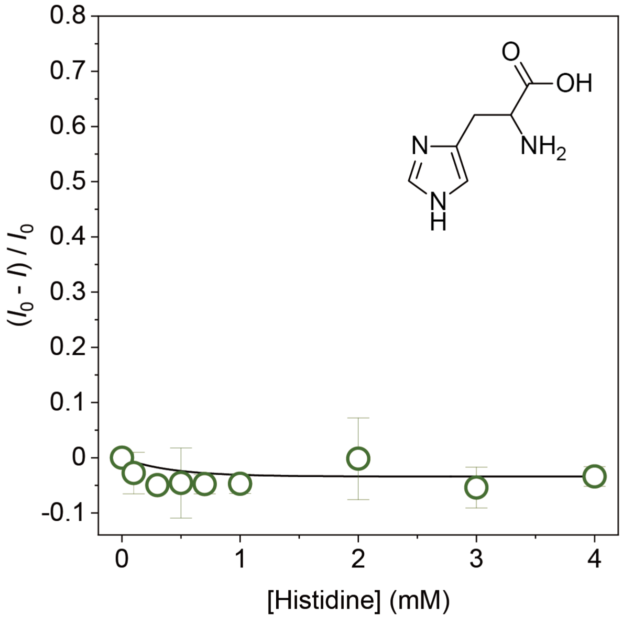

Figure S27 Changes in fluorescence intensity for the polythiophene-ensemble film by histidine at various concentrations in a HEPES buffer solution (100 mM) with TBABr (100 mM) at pH 7.0 at 30°C.

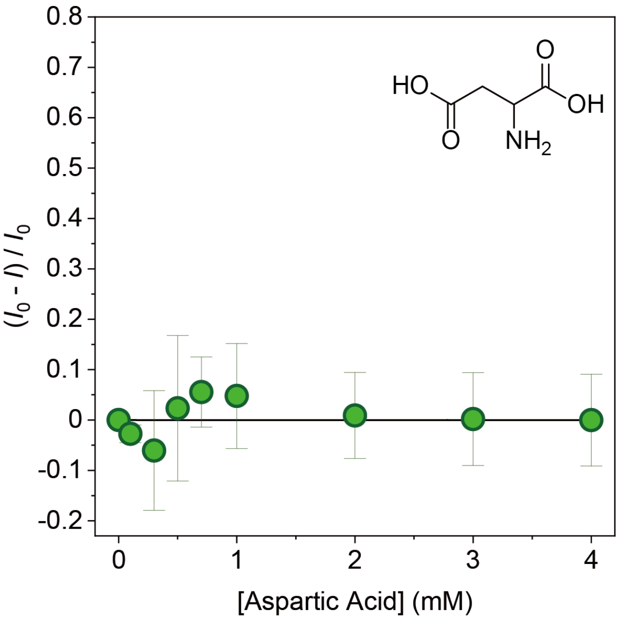

Figure S28 Changes in fluorescence intensity for the polythiophene-ensemble film by aspartic acid at various concentrations in a HEPES buffer solution (100 mM) with TBABr (100 mM) at pH 7.0 at 30°C.

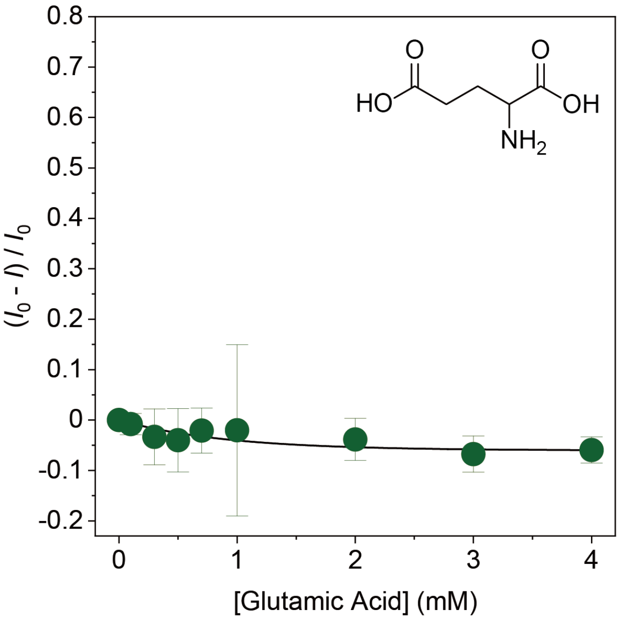

Figure S29 Changes in fluorescence intensity for the polythiophene-ensemble film by glutamic acid at various concentrations in a HEPES buffer solution (100 mM) with TBABr (100 mM) at pH 7.0 at 30°C.

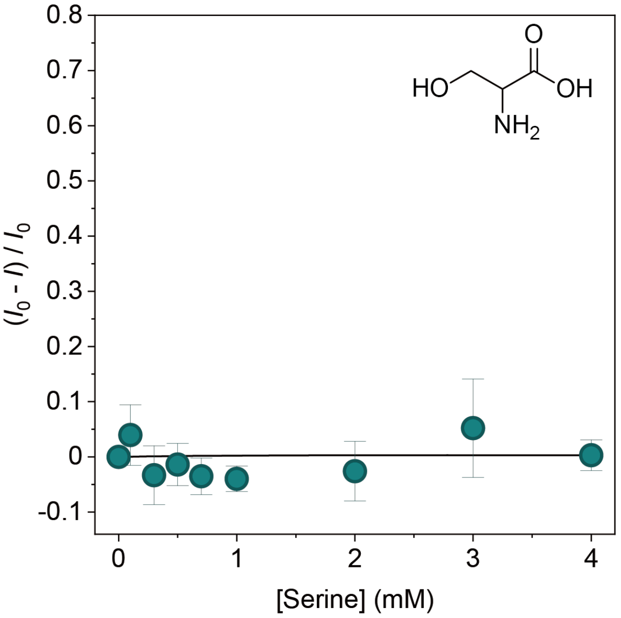

Figure S30 Changes in fluorescence intensity for the polythiophene-ensemble film by serine acid at various concentrations in a HEPES buffer solution (100 mM) with TBABr (100 mM) at pH 7.0 at 30°C.

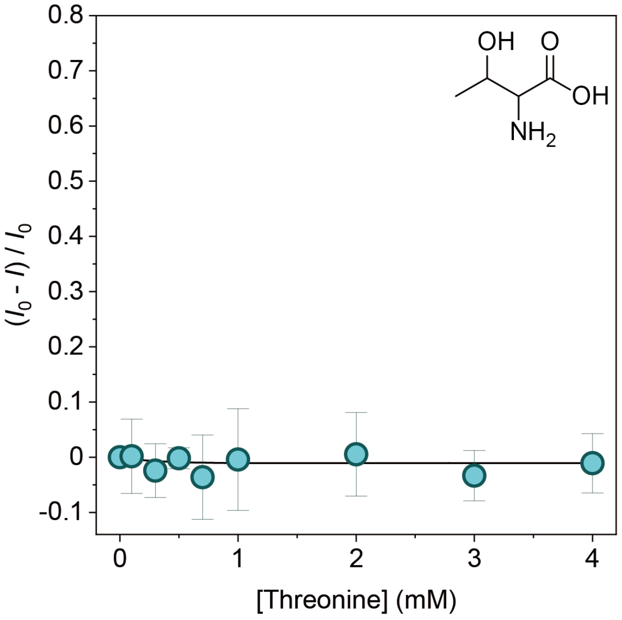

Figure S31 Changes in fluorescence intensity for the polythiophene-ensemble film by threonine acid at various concentrations in a HEPES buffer solution (100 mM) with TBABr (100 mM) at pH 7.0 at 30°C.

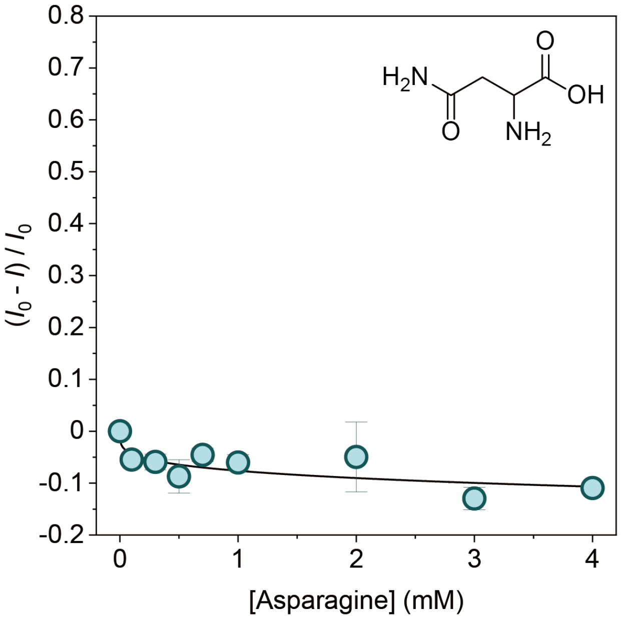

Figure S32 Changes in fluorescence intensity for the polythiophene-ensemble film by asparagine acid at various concentrations in a HEPES buffer solution (100 mM) with TBABr (100 mM) at pH 7.0 at 30°C.

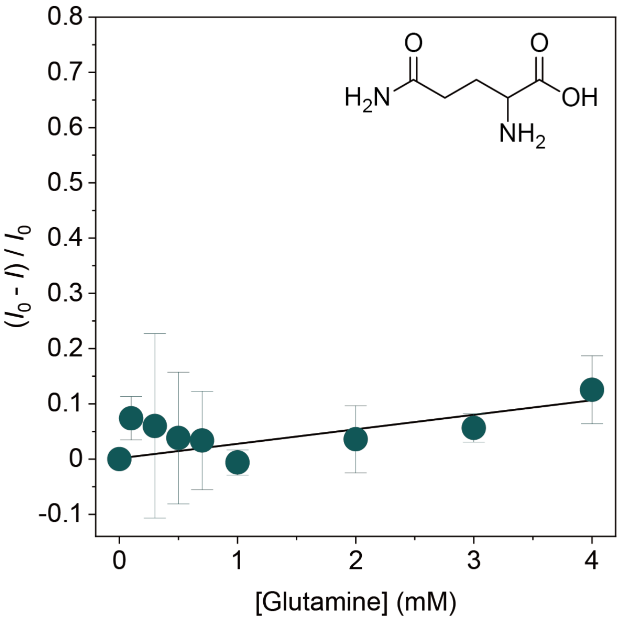

Figure S33 Changes in fluorescence intensity for the polythiophene-ensemble film by glutamine acid at various concentrations in a HEPES buffer solution (100 mM) with TBABr (100 mM) at pH 7.0 at 30°C.

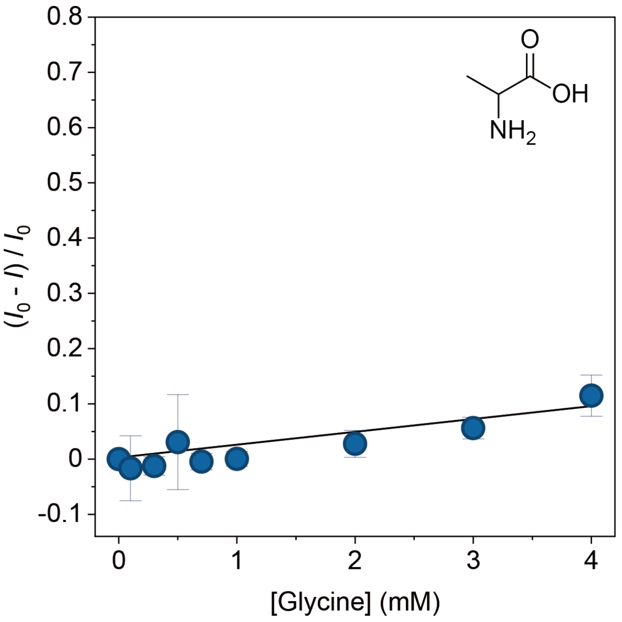

Figure S34 Changes in fluorescence intensity for the polythiophene-ensemble film by glycine acid at various concentrations in a HEPES buffer solution (100 mM) with TBABr (100 mM) at pH 7.0 at 30°C.

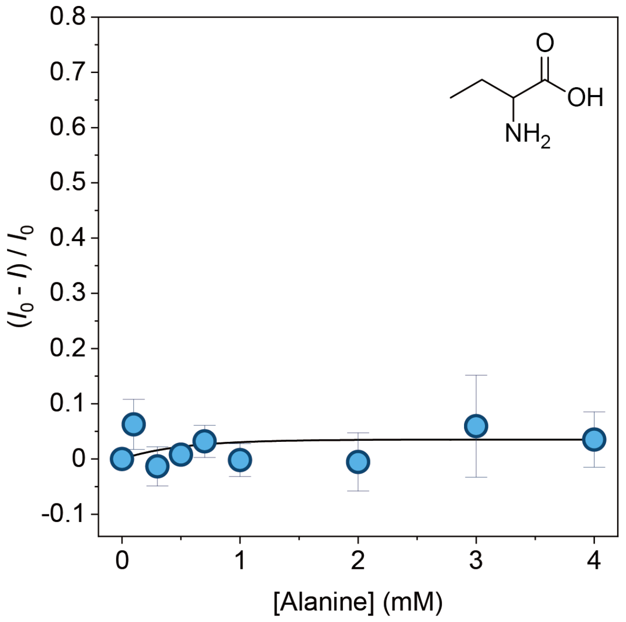

Figure S35 Changes in fluorescence intensity for the polythiophene-ensemble film by alanine acid at various concentrations in a HEPES buffer solution (100 mM) with TBABr (100 mM) at pH 7.0 at 30°C.

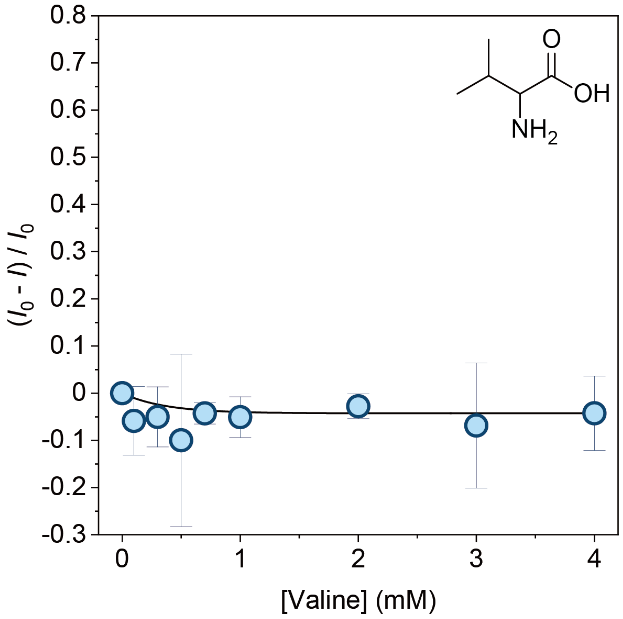

Figure S36 Changes in fluorescence intensity for the polythiophene-ensemble film by valine acid at various concentrations in a HEPES buffer solution (100 mM) with TBABr (100 mM) at pH 7.0 at 30°C.

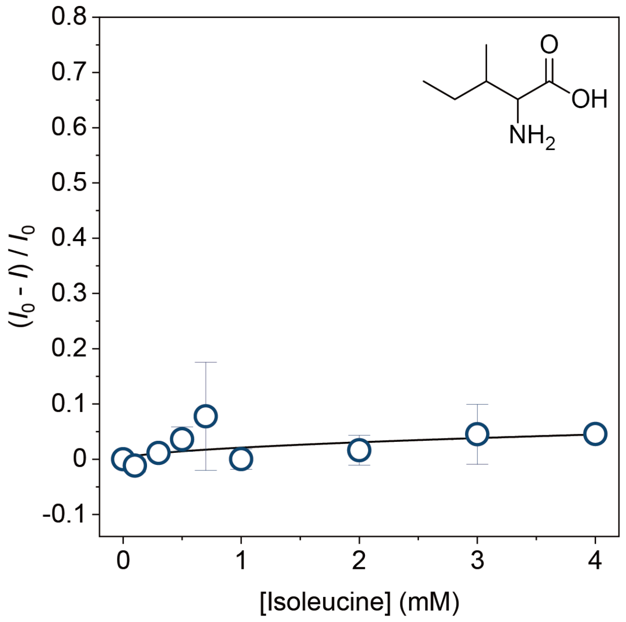

Figure S37 Changes in fluorescence intensity for the polythiophene-ensemble film by isoleucine acid at various concentrations in a HEPES buffer solution (100 mM) with TBABr (100 mM) at pH 7.0 at 30°C.

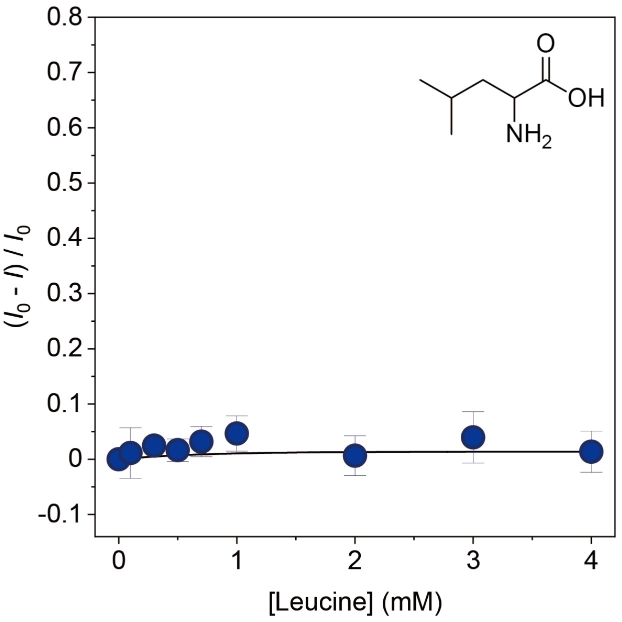

Figure S38 Changes in fluorescence intensity for the polythiophene-ensemble film by leucine acid at various concentrations in a HEPES buffer solution (100 mM) with TBABr (100 mM) at pH 7.0 at 30°C.

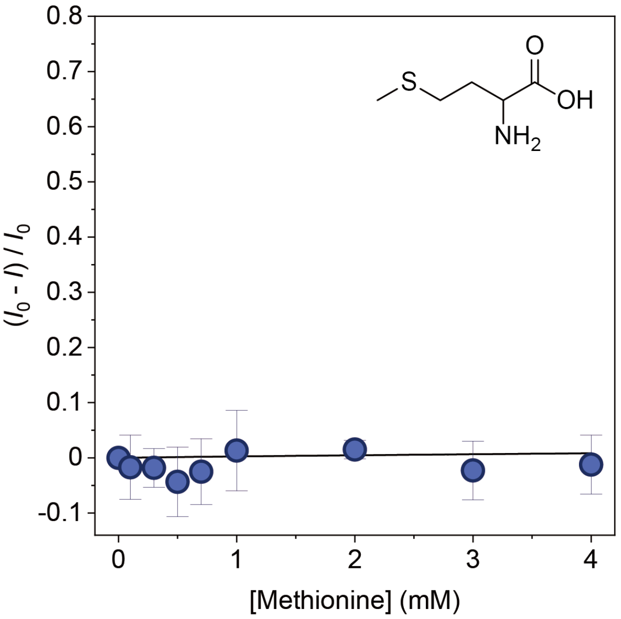

Figure S39 Changes in fluorescence intensity for the polythiophene-ensemble film by methionine acid at various concentrations in a HEPES buffer solution (100 mM) with TBABr (100 mM) at pH 7.0 at 30°C.

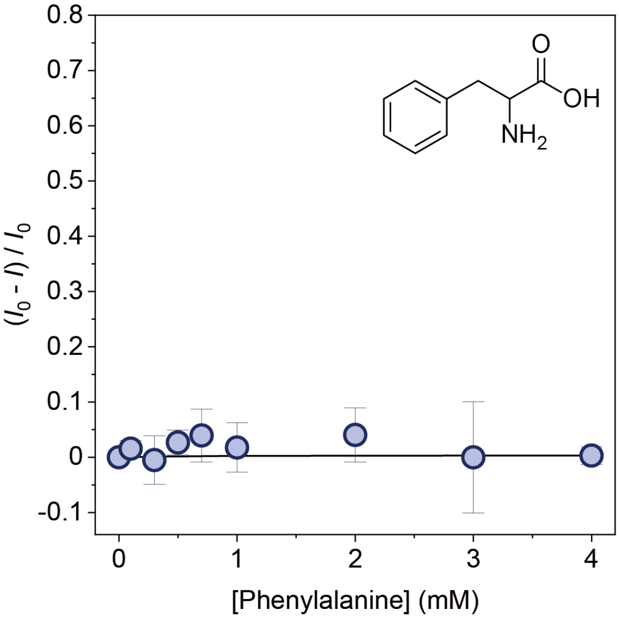

Figure S40 Changes in fluorescence intensity for the polythiophene-ensemble film by phenylalanine acid at various concentrations in a HEPES buffer solution (100 mM) with TBABr (100 mM) at pH 7.0 at 30°C.

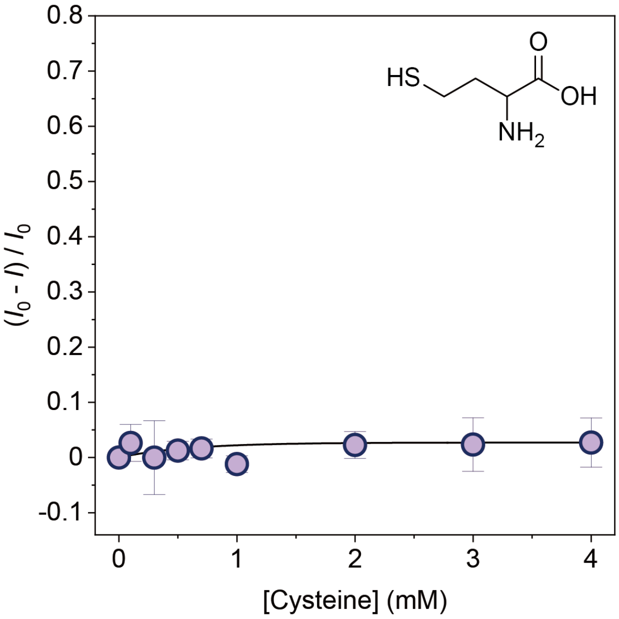

Figure S41 Changes in fluorescence intensity for the polythiophene-ensemble film by cysteine acid at various concentrations in a HEPES buffer solution (100 mM) with TBABr (100 mM) at pH 7.0 at 30°C.

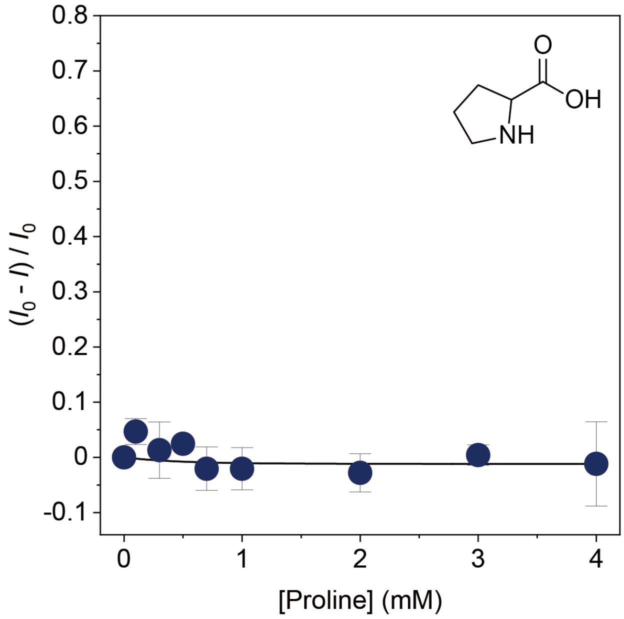

Figure S42 Changes in fluorescence intensity for the polythiophene-ensemble film by proline acid at various concentrations in a HEPES buffer solution (100 mM) with TBABr (100 mM) at pH 7.0 at 30°C.

Comparison of the performance of the fabricated sensor with the reported ones

Table S6. The linear range of the polythiophene-ensemble film (this study) to tobramycin and the reported label-free sensor devices to the same analyte.

Method	Linear range [μM]	References
Fluorescence film sensor based on polythiophene ensemble	0 – 500	This study
Aptamer sensor based on transmission localized surface plasmon resonance	0 – 80	*Anal. Chem.* **2015**, *87*, 5278.[12]
Aptamer sensor based on electrochemical impedance spectroscopy	3 – 72.1	*Biosens. Bioelectron.* **2011**, *26*, 2354.[13]

References

[1] Piest, M.; Zhang, X.; Trinidad, J.; Engbersen, J. F. J. pH-responsive, dynamically restructuring hydrogels formed by reversible crosslinking of PVA with phenylboronic acid functionalised PPO–PEO–PPO spacers (Jeffamines®). *Soft Matter* **2011**, *7*, 11111.

[2] Kim, H. J.; Skinner, M.; Yu, H.; Oh, J. H.; Briseno, A. L.; Emrick, T.; Kim, B. J.; Hayward, R. C. Water Processable Polythiophene Nanowires by Photo-Cross-Linking and Click-Functionalization. *Nano Lett.* **2015**, *15*, 5689.

[3] Na, J. Y.; Kang, B.; Sin, D. H.; Cho, K.; Park, Y. D. Understanding Solidification of Polythiophene Thin Films during Spin-Coating: Effects of Spin-Coating Time and Processing Additives. *Sci. Rep.* **2015**, *5*, 13288.

[4] Lodi, A.; Caselli, M.; Zanfrognini, B.; Cagnoli, R.; Mucci, A.; Parenti, F.; Schenetti, L.; Ponterini, G. Strategies to reduce inter-chain aggregation and fluorescence quenching in alternated multilayers of a polythiophene. *Thin Solid Films* **2008**, *516*, 8731.

[5] Hayashi, S. Highly crystalline and efficient red-emissive π-conjugated polymer film: tuning of macrostructure for light-emitting properties. *Mater. Adv.* **2020**, *1*, 632.

[6] Hu, Z.; Willard, A. P.; Ono, R. J.; Bielawski, C. W.; Rossky, P. J.; Vanden Bout, D. A. An insight into non-emissive excited states in conjugated polymers. *Nat. Commun.* **2015**, *6*, 8246.

[7] Kubo, Y.; Ishida, T.; Minami, T.; James, T. D. Highly Selective Fluoride Ion Detection Based on a Fluorescent Alizarin–*o*-Aminomethylphenylboronic Acid Ensemble in Aqueous MeOH Solution. *Chem. Lett.* **2006**, *35*, 996.

[8] Minamiki, T.; Hashima, Y.; Sasaki, Y.; Minami, T. An electrolyte-gated polythiophene transistor for the detection of biogenic amines in water. *Chem. Commun.* **2018**, *54*, 6907.

[9] Kim, B. S.; Chen, L.; Gong, J.; Osada, Y. Titration Behavior and Spectral Transitions of Water-Soluble Polythiophene Carboxylic Acids. *Macromolecules* **1999**, *32*, 3964.

[10] Hall, Jr., H. K. Correlation of the Base Strengths of Amines. *J. Am. Chem. Soc.* **1957**, *79*, 5441.

[11] Abiman, P.; Wildgoose, G. G.; Crossley, A.; Jones, J. H.; Compton, R. G. Contrasting pKa of Protonated Bis(3-aminopropyl)-Terminated Polyethylene Glycol "Jeffamine" and the Associated Thermodynamic Parameters in Solution and Covalently Attached to Graphite Surfaces. *Chem. Eur. J.* **2007**, *13*, 9663.

[12] Cappi, G.; Spiga, F. M.; Moncada, Y.; Ferretti, A.; Beyeler, M.; Bianchessi, M.; Decosterd, L.; Buclin, T.; Guiducci, C. Label-free detection of tobramycin in serum by transmission-localized surface plasmon resonance. *Anal. Chem.* **2015**, *87*, 5278.

[13] González-Fernández, E.; de-los-Santos-Álvarez, N.; Lobo-Castañón, M. J.; Miranda-Ordieres, A. J.; Tuñón-Blanco, P. Impedimetric aptasensor for tobramycin detection in human serum. *Biosens. Bioelectron.* **2011**, *26*, 2354.
